# Co‐translational protein targeting to mitochondria in the context of co‐translational protein maturation

**DOI:** 10.1002/pro.70682

**Published:** 2026-06-18

**Authors:** Nikita A. Kvasov, Yury S. Bykov

**Affiliations:** ^1^ Quantitative Cell Biology RPTU University Kaiserslautern‐Landau Kaiserslautern Germany

**Keywords:** chaperones, co‐translational import, mitochondria, mRNA localization, NAC, protein targeting, translation

## Abstract

Mitochondria import the majority of their proteins from the cytosol, creating a fundamental challenge: precursor proteins must be synthesized, maintained in an import‐competent state, and delivered to mitochondrial translocases without premature folding or aggregation. While mitochondrial protein import has been considered a post‐translational process, growing evidence shows that a subset of mitochondrial proteins is synthesized in proximity to the organelle. We term this process co‐translational targeting, or local translation. It may lead to direct structural coupling of protein synthesis and import, which we term co‐translational translocation. New approaches, including selective ribosome profiling, proximity labeling, and RNA imaging, reveal that mitochondrial mRNA localization is highly dynamic and can be driven by both RNA‐based and translation‐dependent mechanisms. In contrast to the well‐defined signal recognition particle pathway at the endoplasmic reticulum, mitochondrial targeting appears to rely on more flexible mechanisms shaped by nascent‐chain properties, translation elongation, and coding‐sequence features beyond the targeting signal. We discuss how these processes may support mitochondrial biogenesis and proteostasis while also creating vulnerabilities associated with ribosome stalling and precursor quality control. Together, recent findings position mitochondrial protein targeting as an integral part of cellular protein biogenesis and highlight key open questions in the coordination of translation and organelle function.

## 1 INTRODUCTION

To function properly, the protein needs to be correctly folded and correctly localized. The localization address for each protein is encoded in its primary sequence and recognized by the protein targeting systems of the cell. They are particularly complex in eukaryotic cells that have many membrane‐bound organelles. How protein maturation performed by ribosomes and chaperones is coordinated with protein transport remains poorly understood.

The two major protein delivery destinations in the cell are the endoplasmic reticulum (ER) and mitochondria. The ER features the signal recognition targeting particle (SRP) pathway that can directly link translation and protein translocation across the membrane. Membrane‐bound ribosomes produce a familiar textbook view of the rough ER. Much less is known about the links between translation and translocation of mitochondrial proteins. Early works from the Butow laboratory showed that ribosomes can associate with mitochondrial membrane and predominantly synthesize mitochondrial proteins (Kellems et al., 1974, 1975; Kellems & Butow, 1972). At that time, when the major targeting pathways were yet to be fully uncovered, a natural idea was that mitochondrial protein import may rely on co‐translational pathway conceptually like the ER‐targeting pathway. This idea did not agree well with the view of mitochondrial import as mostly post‐translational (Eilers & Schatz, 1986; Neupert, 1997). Discussing this, Butow and colleagues suggested that the two import modes, post‐ and co‐translational, might be not mutually exclusive, but can compete or co‐exist in one cell (Ades & Butow, 1980a). Following research supports this idea. Different approaches showed that specific subset of proteins is synthesized next to mitochondria, this we term *co‐translational targeting*, or *local translation*. There is much less evidence supporting that such local translation is mechanistically coupled to translocation across the membrane, a process we term *co‐translational import*. In this review we will focus on the recent methodological advances and consider the possible mechanisms and biological roles of co‐translational targeting and import into mitochondria in the context of protein biogenesis.

## 2 PROTEIN BIOGENESIS IN THE ORGANELLE CONTEXT

**FIGURE 1:**
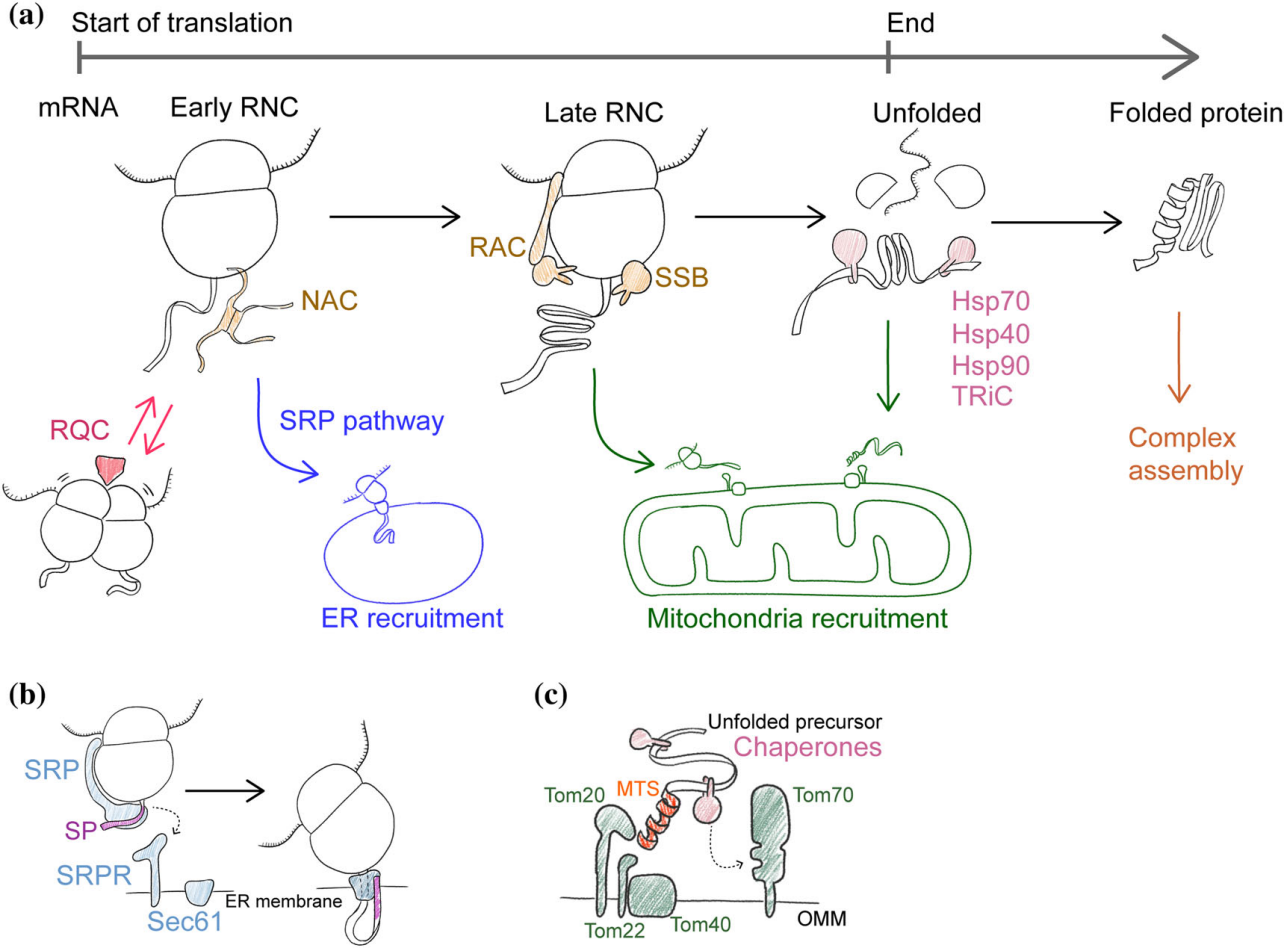
ER and mitochondria targeting pathways in the context of protein maturation. (a) General co‐translation chaperones like nascent chain associated complex (NAC), ribosome‐associated complex (RAC), and co‐translational Hsp70 (SSB) can bind the ribosome‐nascent chain complex (RNC) throughout the translation process. The finished unfolded nascent chain is bound by post‐translational chaperones like Hsp70, Hsp40, Hsp90, and TRiC that promote its folding. Translation pauses can be recognized and resolved by the ribosome quality control (RQC). The signal recognition particle (SRP) pathway acts early during translation to deliver RNC to the ER (blue lines). Most mitochondrial proteins are delivered to mitochondria after translation is finished, but some are recruited at the RNC stage. Cytosolic protein complex assembly can start before translation is finished. (b) SRP acts by binding the signal peptide (SP) after it emerges from the ribosome. SRP halts translation and delivers the RNC to the ER surface by binding the SRP receptor (SRPR). Then the ribosome binds the Sec61 channel and translation is resumed. (c) Mitochondria mostly import unfolded proteins bound by chaperones. The N‐terminal mitochondrial targeting signal (MTS) binds the outer mitochondrial membrane (OMM) receptors Tom20 and Tom22 and then the protein is translocated through the pore‐forming subunit Tom40. The receptor Tom70 can bind chaperones promoting protein import.

The path from mRNA to a functional folded protein is complex, even without organelles (Figure 1a). The first step is translation initiation that is considered to be the main regulatory checkpoint (Brito Querido et al., 2024). After initiation succeeds, elongation follows, and the nascent chain starts to emerge from the ribosome exit tunnel. The resulting ribosome–nascent chain complex (RNC) is recognized by conserved co‐translational factors that ensure timely modification and proper folding of the newly synthesized protein. One of the first factors that can engage the ribosome even before the nascent chain is synthesized is nascent‐chain associated complex (NAC) (Wang et al., 1995; Wiedmann et al., 1994). NAC orchestrates co‐translational processing of the nascent chain N‐terminus involving initiator methionine removal and nascent chain acetylation (Gamerdinger & Deuerling, 2024). NAC remains associated with the ribosome during elongation and also acts as a chaperone shielding exposed hydrophobic regions and promoting folding (Lee et al., 2026; Santos et al., 2026). Another factor that binds RNCs is ribosome‐associated complex (RAC) (Gautschi et al., 2001). RAC can bind the nascent chain and promote its handover to Ssb (Ssb1 and Ssb2 in yeast), a ribosome‐associated Hsp70 family member and a general co‐translational chaperone (Döring et al., 2017; Willmund et al., 2013; Zhang et al., 2020). After translation is completed, unfolded protein is handed over to the network of cytosolic chaperones such as Hsp70, Hsp90, TRiC and their co‐chaperones for folding (Balchin et al., 2016). Protein folding and complex assembly can also start co‐translationally (Bertolini et al., 2021; Kramer et al., 2019; Mallik et al., 2025; Shiber et al., 2018). Complex dynamics of co‐translational folding and assembly may be regulated by programmed translation pauses (Aguilar Rangel et al., 2024). The rate of elongation is not uniform along the transcript. It is determined by codon optimality, codon combinations and tRNA availability (Schuller & Green, 2018). Non‐optimal codons can induce ribosome states that are recognized by the CCR4‐NOT complex that deadenylates translated mRNA thus regulating its stability based on codon composition (Zhu et al., 2024). Translation pauses caused by mutations, mRNA damage, or other stress factors can result in prolonged ribosome stalls. These stalls cause ribosome collisions and trigger rescue mechanisms such as non‐stop and no‐go mRNA decay, and ribosome quality control (RQC) (Collart & Weiss, 2020; Inada & Beckmann, 2024). When rescue systems are overwhelmed, stalled ribosomes can trigger stress signaling and cell death (Nanjaraj Urs et al., 2024; Wu et al., 2020). Thus, translation elongation emerges as another important step in regulating the expression level, folding, and fate of the newly synthesized protein on par with translation initiation.

How does organelle protein biogenesis fit into this scheme (Figure 1a)? Nucleus and peroxisomes can import folded proteins while ER and mitochondria require unfolded proteins for translocation through their membranes. For this reason, targeting pathways to ER and mitochondria tap into protein synthesis before the folding and complex assembly is completed (Figure 1a). Signal recognition particle (SRP) pathway that delivers proteins to the ER is a prototypic example of a co‐translational mechanism that acts early in protein biogenesis (Figure 1b). SRP is a ribonucleoprotein complex that recognizes an N‐terminal targeting signal (signal peptide, SP) when it emerges from the ribosome (Akopian et al., 2013). Then the RNC–SRP complex docks to the SRP‐receptor, an ER membrane protein. SRP dissociates, the ribosome binds Sec61 translocon and the nascent chain translocates into the ER (Voorhees et al., 2014). These events happen relatively early during the translation time course (Chartron et al., 2016; Jan et al., 2014; Schibich et al., 2016). ER proteins that have targeting signals away from the N‐terminus do not depend on the SRP pathway and can be delivered post‐translationally (Ast et al., 2013; Aviram et al., 2016).

Mitochondrial proteins can be recruited to the outer mitochondrial membrane (OMM) at different stages of translation—this is the subject of this review (Figure 1a). Nevertheless, all the intermembrane space and matrix proteins enter mitochondria as unfolded precursor proteins through the translocase of the outer membrane (TOM) complex (Dekker et al., 1998; Hill et al., 1998) (Figure 1c). A core TOM complex contains a pore‐forming subunit Tom40, smaller proteins Tom5, Tom6, and Tom7, and a stably bound receptor Tom22 (Araiso et al., 2019; Dekker et al., 1998; Shiota et al., 2015). The two other receptors Tom20 and Tom70 are loosely associated with the core TOM complex. The main function of Tom20 and Tom22 is to recognize the mitochondrial targeting signal (MTS) in a translocated protein (Brix et al., 1997; Yamano et al., 2008). Most of the proteins destined to the inner mitochondrial membrane (IMM) and the matrix have an N‐terminal MTS, also called presequence (Vögtle et al., 2009). MTS is usually a 10–100 amino acid long peptide with a propensity to form a positively charged amphipathic alpha‐helix (von Heijne, 1986). A notable group of substrates without presequence are IM metabolite carriers that have internal targeting signals (Nauerz et al., 2025). The Tom70 receptor can recognize both N‐terminal and internal targeting signals (Backes et al., 2018; Brix et al., 1997). Another important function of this receptor is binding cytosolic chaperones such as Hsp70 that deliver unfolded precursors to the TOM (Backes et al., 2021; Young et al., 2003). While the translocation mechanisms for many types of mitochondrial proteins are well studied, it remains relatively poorly understood how each of them is delivered to the OMM with the help of co‐translational targeting, or post‐translational chaperones (Bykov et al., 2020).

## 3 THE METHODS TO DEFINE THE SUBSTRATES OF CO‐TRANSLATIONAL IMPORT AND LOCAL TRANSLATION

To unravel the mechanism of the targeting pathway, one needs to find its substrate spectrum. Co‐translational targeting and import imply that mRNA would be translated next to the targeting destination of the protein. Thus, many works aimed to determine the localization of mRNAs encoding mitochondrial proteins. The main approaches applied to determine mRNA localization were sequence‐based, like organelle‐associated polysome fractionation and proximity labeling, or imaging‐based (Table 1). The potential substrate ranges derived from these works showed remarkable variability depending on the method or conditions studied.

**TABLE 1:** High‐throughput and medium‐throughput studies of mRNA localization to mitochondria, the methods they used, and their main findings (in chronological order).

References	Organism	Method	Details	Result	Additional information
Marc et al. (2002)	Yeast	Organelle fractionation, polysome purification, microarray	Spheroplasts, CHX for 10 min before lysis, crude mitochondria fraction compared to cytosolic polysome fraction	Found 467 associated mRNAs with MLR Score >80.	The activity of several UTRs confirmed; localization linked to the bacterial origin of genes
Sylvestre et al. (2003)	Yeast	Same as above	20 min CHX before lysis; improved normalization of expression	95% of mitochondrial protein mRNAs are quantified with the new MFI score	
Garcia et al. (2006)	Yeast	Same as above with qPCR‐based mRNA quantification	Additional FISH verifications	Quantified 112 mRNAs; ATP2‐4 confirmed by FISH	
Saint‐Georges et al. (2008)	Yeast	Same as above	Tested the effect of CHX and puromycin before lysis; tested *puf3∆*	480 associated RNAs classified based on Puf3 dependence	CHX reduced RNA association
Eliyahu et al. (2010)	Yeast	Direct mRNA extraction from cytosolic or mitochondrial fraction	Used CHX before cell lysis; compared *tom20∆* and puf3∆ mutants	Tom20 deletion reduces mRNA association	Northern blot verification of ACO1 and SHM1 mRNA localization
Gadir et al. (2011)	Yeast	MS2L‐tagged mRNA imaging	Studied 20+ mRNAs; tested Puf3 and TOM dependence	Most mRNA localizations depend on TOM; limited role of Puf3	
Williams et al. (2014)	Yeast	Selective ribosome profiling; OMM‐associated ribosomes marked by proximity biotinylation	Biotin pulse for 2 min; compared with and without CHX before pulse	Relatively small set of OMM‐associated mRNAs; the set changes after CHX treatment	
Luo et al. (2025)	Human cell culture	Same as above	Biotinylation is triggered by a pulse of light	Short mRNAs associate via RBPs; long mRNAs are recruited late in translation	Recruitment of individual mRNAs was measured
Zhu et al. (2025)	Human cell culture	Selective ribosome profiling; co‐purification with TOM	Tested the effect of CHX and NAC downregulation	Long mRNAs are recruited long in translation	Recruitment of individual mRNAs was measured

### 3.1 Sequence‐based approaches

**FIGURE 2:**
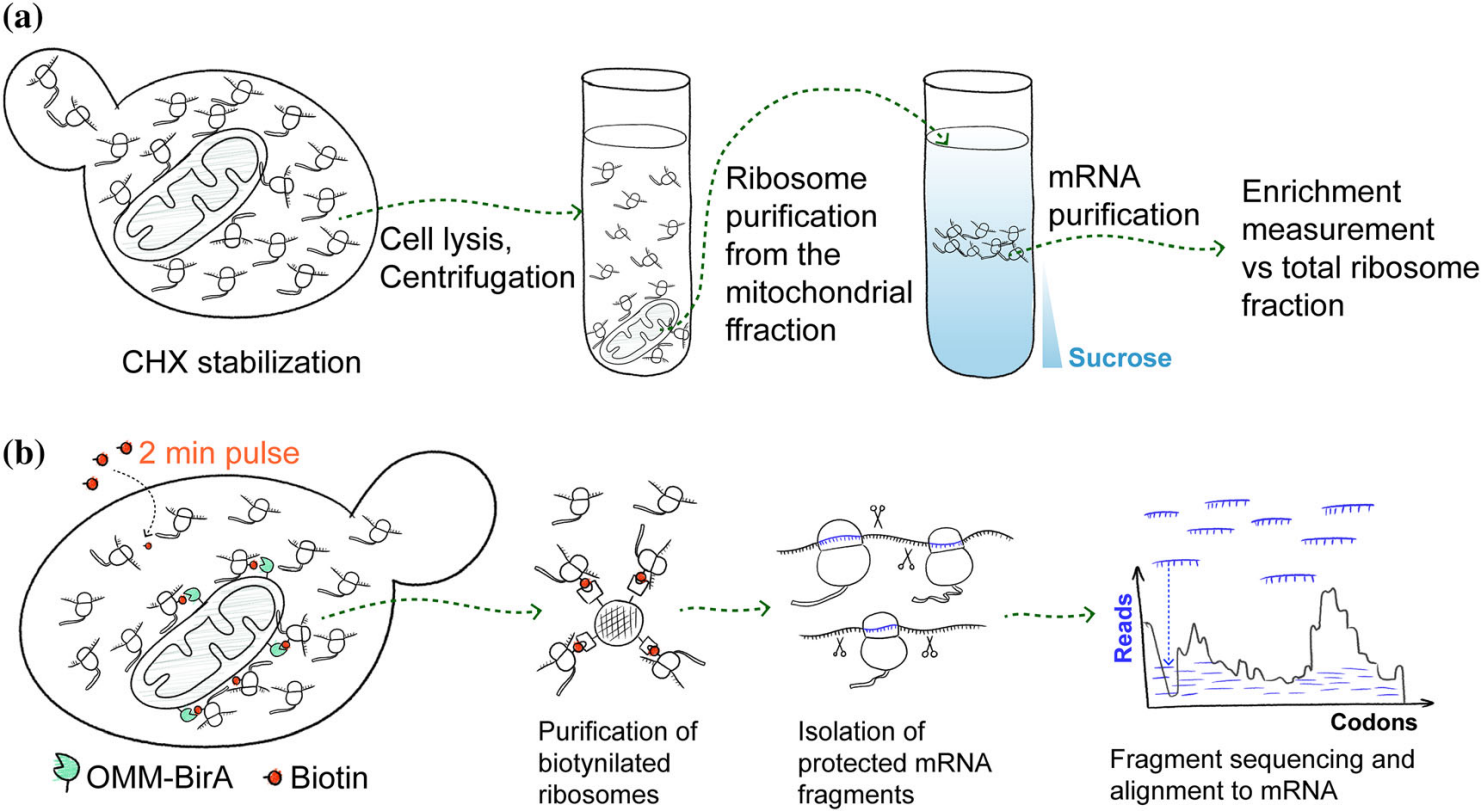
Sequence‐based methods to study local translation. (a) Purification‐based methods typically rely on stabilization of mitochondria‐associated translating ribosomes by cycloheximide (CHX), mild cell lysis, and purification of the mitochondria by differential centrifugation. The resulting fractions are used to purify mRNAs and compare their abundance between the samples by sequencing, qPCR, or microarrays. (b) Proximity biotinylation methods rely on biotin ligase BirA placed on the outer membrane (OMM) that biotinylates mitochondria‐associated ribosomes in living cells over a short time period. Biotinylated ribosomes are purified from the total ribosomal fraction on streptavidin affinity resin and treated with RNAse to digest the mRNAs. Undigested mRNA fragments protected by ribosomes are sequenced and aligned to gene sequences. The read density on biotinylated ribosomes compared to total ribosomes shows when during translation the RNC is recruited to the OMM.

The first high‐throughput study of mRNAs translated in the proximity of mitochondria adapted the protocol developed by Ades and Butow (1980a, 1980b): budding yeast (hereafter, just “yeast”) cells were incubated in the presence of cycloheximide (CHX) to stabilize polysomes, then the cells were gently lysed and a crude mitochondrial fraction was separated from the rest of the lysate. Then polysomes were purified from the total lysate and from the mitochondrial fraction (Figure 2a). The concentrations of mRNAs were compared between these two samples using micro‐array hybridization or qPCR to determine the enrichment ratio (Garcia et al., 2006; Marc et al., 2002; Saint‐Georges et al., 2008; Sylvestre et al., 2003). The enrichment ratio was used to determine the mRNA localization score that was calculated relative to mtDNA‐encoded RNAs. Depending on the exact protocol and the method of score calculation and threshold selection, a few hundred yeast mRNAs were assigned as locally translated. For example, one of the later works defined 480 yeast mRNAs as locally translated out of a total of 794 mRNAs encoding mitochondrial proteins (Saint‐Georges et al., 2008). The common features of these mRNAs were that they encoded relatively large proteins, many of which were components of respiratory complexes of bacterial origin, such as ATP‐synthase subunits Atp1, Atp2, and Atp3 (Sylvestre et al., 2003).

To avoid the possible artifacts that may be caused by cell fractionation, selective ribosome profiling (SeRP) was adapted to study local translation in yeast (Ingolia et al., 2019). The method relies on fast and specific biotin‐ligase BirA that biotinylates only its acceptor peptide, AviTag, when positioned in proximity (Beckett et al., 1999). BirA was placed on the outer membrane of mitochondria by fusing it with protein Om45, while AviTag was fused to a protein of the large ribosomal subunit (Figure 2b). The cells were grown in low‐biotin media and pulsed with high concentrations of biotin for 2 min—a period needed to make an average protein—to only biotinylate the ribosomes in the vicinity of the OMM. Biotinylated ribosomes were then purified from the total ribosomes and the mRNA fragments protected by the assembled subunits (footprints) were sequenced (Williams et al., 2014). For each mRNA, the enrichment level in the proximal ribosomes relative to total was calculated. Unlike the previous polysome purification approach that requires the ribosome–mitochondria interaction to hold throughout the purification procedure, proximity biotinylation may also detect weak or temporary interactions. Possibly, this was the reason why the set of locally translated proteins detected by SeRP was different from the earlier purification‐based studies. The SeRP data revealed that a relatively small number of proteins, many of which were destined for the inner membrane, were locally translated (Williams et al., 2014). Examples of such yeast proteins are translocase of the inner membrane subunit Tim50, protease Yme1, and membrane transporter Mdl2. Almost all of these proteins were longer than 180 amino acids. Interestingly, blocking protein synthesis by CHX a few minutes before biotin pulse made more matrix protein‐encoding mRNAs enriched at the OMM (Saint‐Georges et al., 2008). The mRNAs recruited to the OMM in a CHX‐dependent way included *ATP1*, *ATP2*, and *ATP3*, all of which, according to SeRP, have low mitochondrial association without CHX treatment. This finding indicates that co‐translational targeting is highly dynamic and can be dramatically affected by experimental conditions.

Ribosome profiling was also applied to investigate co‐translational targeting and import in cultured human cells. The two recent works used different approaches for enriching mitochondria‐associated ribosomes. One work employed light‐inducible BirA fused to an OMM protein transmembrane domain and AviTag‐tagged ribosomes (Luo et al., 2025), and another work directly isolated TOM‐bound ribosomes using affinity purification (Zhu et al., 2025). Both studies revealed similar subsets of long (>200 residues) inner membrane and matrix proteins translated next to the outer membrane, or in a TOM‐bound way. Some of these substrates were conserved between yeast and humans (Luo et al., 2025; Williams et al., 2014). The BirA‐based study additionally identified a subset of shorter (<200 residues) substrates. Both studies used mapping of ribosome footprints to the mRNA sequence to estimate the “timing” (in synthesized amino acids, not seconds) of RNC recruitment to the outer membrane and came to the same conclusion. All of the “long” substrates were recruited relatively late, after more than 200 residues were synthesized (Luo et al., 2025; Zhu et al., 2025). The small subset of short substrates was recruited relatively early or even before translation start suggesting an RNA‐dependent mechanism (see next section) (Luo et al., 2025). The analysis of long substrates revealed that proteins containing folded domains with certain characteristics tend to be preferentially locally translated, but no specific functional category or recruitment signal was identified (Zhu et al., 2025).

Another high‐throughput method used to study mRNA‐localization in mammalian cells was APEX‐seq (Fazal et al., 2019). It relies on engineered ascorbate peroxidase (APEX2) that can conjugate biotin to surrounding proteins and mRNAs. In this work, APEX2 was targeted to different cellular locations, including the OMM, and the biotinylated RNAs were purified and sequenced. A set of mitochondrial protein‐encoding mRNAs was found next to the OMM. Similarly to SeRP data in yeast, CHX treatment had a strong effect, increasing the localization scores of mRNAs with high MTS prediction scores. Treatment with puromycin that disassembles RNCs helped to define a subset of mRNAs that localize to OMM in a translation‐independent manner. A machine learning approach was able to predict the localization of such mRNAs revealing that the 3′ untranslated region (UTR) is particularly important for such prediction. APEX2 fused to mammalian TOMM20 and TOMM70 was also used to define the RNA‐binding proteins (RBPs) in the proximity of these receptors (Akram et al., 2026). The work revealed a greater role of TOMM20 in retaining protective RBPs next to the mitochondria under stress.

### 3.2 Imaging‐based approaches

Imaging is an alternative way to visualize mRNA localization and investigate local translation. The two main approaches are fluorescent in situ hybridization (FISH) and aptamer tags. FISH employs oligonucleotides conjugated to a dye that are hybridized to RNAs in fixed and permeabilized cells. The advantage of this method is that native, unmodified mRNAs can be visualized. Aptamer tags are nucleotide sequences that can fold into three‐dimensional structures as single‐stranded mRNA and bind proteins or small molecules. One of the most commonly used aptamers is MS2 stem loop (MS2L) derived from MS2 phage (Pichon et al., 2020). One transcribed MS2L repeat can bind MS2 phage coat protein (CP). Typically, one mRNA is tagged with 12–24 MS2L repeats and co‐expressed with CP that is fused to 4–6 copies of GFP. This high number of fluorophores allows us to visualize single mRNA molecules. The advantage of this approach is that it works in living cells and is cheaper than FISH. However, aptamer tags can affect mRNA localization and turnover. To control that, FISH is often used to verify data obtained by aptamer tags. Both imaging methods remain relatively low throughput. While there are approaches to multiplex FISH by combining it with sequencing or iterative labeling, those were not systematically applied to localize mRNAs in relation to mitochondria (Mi et al., 2025; Xia et al., 2019).

Several yeast mRNAs were characterized by imaging in the context of protein targeting. For example, FISH was used to confirm mitochondrial localization of *ATP3* and *ATP4* mRNAs discovered by polysome purification (Garcia et al., 2006). MS2L‐tagging was used to investigate the mechanism of *ATP2* localization (Margeot et al., 2002) and to survey localization of more than 20 other mRNAs encoding mitochondrial proteins (Gadir et al., 2011). Both studies showed high co‐localization of *ATP2* mRNA with mitochondria. However, the challenges in quantifying spot‐like mRNA signals in images make comparisons between studies difficult. A work that employed live imaging of MS2L‐tagged mRNAs showed that yeast *TIM50* mRNA had consistently high mitochondrial localization but *ATP1* and *ATP3* mRNAs changed their association with mitochondria depending on growth conditions: co‐localization increased in respiratory media compared to fermentative media or after CHX treatment (similarly to SeRP data). These results emphasize that individual imaging studies can be complementary to high‐throughput characterization (Tsuboi et al., 2020).

Both approaches for RNA imaging do not report on the status of protein synthesis. For this, additional methods such as SunTag system can be used (Tanenbaum et al., 2014). SunTag comprises an array of epitope tags inserted in the coding sequence and co‐expressed together with GFP‐tagged nanobodies against this epitope. Once synthesized, the tag array binds nanobodies and produces a bright punctum marking a translated mRNA. Tagging of mitochondrial fission factor (MFF) with SunTag array was used to show that this protein is locally produced at the sites of mitochondrial fission in neurons under control of FMRP protein (Fenton et al., 2024). A similar approach was used to show that PINK1 protein is translated at the contact of ER and lysosomes and that this is required to hand over PINK1 precursor to neuronal mitochondria (Hees, Segura, et al., 2024).

Both sequencing and imaging data show that mRNAs of imported mitochondrial proteins can be translated next to the organelle. The exact list of these substrates is hard to define since the distribution of mRNAs depends on the growth conditions and experimental setup. Compared to the ER protein import where a hydrophobic transmembrane domain or signal peptide close to the N‐terminus is predictive of SRP‐dependence (Aviram et al., 2016), no such common features were identified among locally translated mitochondrial proteins. The comparisons made in high‐throughput studies and individual mRNA investigations nevertheless suggest that there exist selectivity mechanisms that promote local translation of some proteins.

## 4 THE MECHANISMS OF CO‐TRANSLATIONAL TARGETING

Despite many years of research, there is no clear vision of how co‐translational targeting and import of mitochondrial proteins might be organized, and how many pathways exist. Three possible stages were considered in the literature: mRNA recruitment before translation initiation that results in local translation; nascent chain or ribosome tethering to mitochondria (co‐translational targeting); and nascent chain handover to the TOM complex (co‐translational import). The stages may not necessarily follow each other in this strict order.

### 4.1 mRNA recruitment

In this article, we discriminate between RNA‐dependent mRNA localization that happens in a translation‐independent way and nascent‐chain dependent localization that we term co‐translational targeting and discuss in Section 4.2. Localization of mRNAs that does not depend on translation is a well‐known phenomenon in complex cell types such as neurons (Bourke et al., 2023). It is maintained by different families of RBPs that recognize RNA sequence motifs directly and bring mRNA to the cellular destination. Such proteins also play an important role in regulating mRNA stability and translation in this way connecting mRNA targeting with local translation and protein import. RBPs utilize both conserved common motifs to manage large functional groups of transcripts as well as individualized recognition patterns for specific structural or stress‐related mRNAs (Sharma & Fazal, 2024). Here, we briefly review several examples of RBPs that might be involved in regulating local translation at the mitochondrial surface and can affect protein import. For a more detailed review of RBP regulatory activities related to mitochondrial function we refer the reader to more comprehensive reviews (Sharma & Fazal, 2024; Zilio et al., 2025).

In yeast, one of the most studied RBPs in the context of mitochondrial protein import is Puf3 (Gadir et al., 2011; Gerber et al., 2004; Saint‐Georges et al., 2008). It belongs to the Pumilio‐homology domain family (Nishanth & Simon, 2020; Quenault et al., 2011). To recognize its targets, Puf3 utilizes distinct 8‐nt motifs (UGUANAUA) in the 3′‐UTR of the mRNAs (Sharma & Fazal, 2024). Puf3 is a peripheral membrane protein that localizes to the cytosolic face of the OMM (García‐Rodríguez et al., 2007). The range of its targets may include few hundred mRNAs, not all of which encode mitochondrial proteins (Gerber et al., 2004). The effect of Puf3 on its targets can be bidirectional. It was found to bind mRNAs encoding mitochondrial proteins, promote their localization to mitochondria and translation (Lee & Tu, 2015; Miller et al., 2014). It may also assist Tom20 in anchoring mRNAs that are recruited in a translation‐dependent manner (Eliyahu et al., 2010). Puf3 can also promote deadenylation and degradation of such mRNAs (Miller et al., 2014; Olivas & Parker, 2000). The switch in Puf3 activity is regulated by carbon source, phosphorylation, and binding partners (Bulmash et al., 2023; Hayashi et al., 2022; Lee & Tu, 2015). It was also shown that the differences between yeast genetic backgrounds such as S228C and CEN.PK can affect the activity of Puf3 on fermentative or respiratory media (Caballero et al., 2025). Mammalian Puf3 homologs PUM1 and PUM2 were not yet implicated in regulating mitochondrial mRNAs. To sum up, Puf3 has a multifaceted role in posttranscriptional regulation of respiratory metabolism via several different pathways. Whether it can mechanistically link mRNA targeting to protein import into mitochondria remains poorly understood.

A conserved RBP that gained attention as a stress‐related spatial regulatory factor is mammalian clustered mitochondrial homolog (CLUH), named Clu1 in yeast (Cox & Spradling, 2009; Fields et al., 1998). It can bind and promote translation of mitochondrial protein‐encoding RNAs (Gao et al., 2014; Sen & Cox, 2016; Zaninello et al., 2024). In yeast, Clu1 forms granules close to mitochondria when glucose is depleted and a respiratory carbon source is present (Miller‐Fleming et al., 2025). These condensates require translated RNAs and ribosomes for their assembly and are distinct from other stress‐induced foci. The mechanism of substrate mRNA selection or mitochondrial binding of Clu1 is unknown.

In animals, several specific RBPs can recruit mRNAs to the surface of mitochondria. For example, in neurons *Pink1* mRNA interacts with Synaptojanin 2 (SYNJ2) via its RNA‐binding motif (Harbauer et al., 2022). This complex is recruited to the mitochondrial outer membrane by Synaptojanin 2 binding protein (SYNJ2BP; also called OMP25) whose N‐terminal domain binds SYNJ2 and C‐terminal domain contains OMM anchor (Harbauer et al., 2022; Nemoto & De Camilli, 1999). This results in local production of PINK1 protein promoting mitophagy (Harbauer et al., 2022). A more general regulator of mitochondria‐associated translation is AKAP1 protein and its ortholog MDI in Drosophila. This is a scaffold protein that binds cAMP‐dependent protein kinase (AMPK) via the PKA helix and mitochondria via the N‐terminal domain (Huang et al., 1997; Lin et al., 1995). The KH‐Tudor domain of AKAP1 likely mediates its mRNA interactions by binding RBPs called LARP4 and PABC1 (Gabrovsek et al., 2020; Ginsberg et al., 2003; Rogne et al., 2006). The *Drosophila* MDI also binds an RBP Larp that is similar to LARP4 (Zhang et al., 2016). LARP4 may be an AMPK substrate and bind mRNAs when phosphorylated (Gabrovsek et al., 2020). PABC1 and LARP4 bind multiple mRNAs, and how the AKAP1‐associated mRNA selection is achieved remains to be determined (Lewis et al., 2024; Passmore & Coller, 2022) Pull‐down and sequencing of mRNAs bound to the KH‐Tudor domain of human AKAP1 showed preference for the mRNAs encoding citric acid cycle enzymes and components of the respiratory complex II. Notably, this includes succinate dehydrogenase, which bridges these two pathways by functioning in both the citric acid cycle and the electron transport chain (Gabrovsek et al., 2020). These results are supported by the SeRP analysis of mitochondria‐associated translation that revealed that in human cells short mRNAs encoding respiratory chain complex subunits are the targets of AKAP1 (Luo et al., 2025). Structure–function analysis of individual locally translated proteins NDUFB9 and COX7A2 revealed that mRNA recruitment depended not only on the UTRs but also on the presence of introns in the original DNA sequence (Luo et al., 2025). This points to a yet unknown mechanism that can discriminate properly spliced transcripts for their mitochondrial targeting and local translation. OMM‐enriched ribosome footprints of the potential AKAP1 substrates map to these mRNAs over their entire length supporting the idea that AKAP1 promotes mRNA recruitment and translation in the vicinity of mitochondria.

### 4.2 Translation‐dependent localization

While RBP‐mediated mRNA targeting followed by local translation can lead to protein import, the mechanistic links are still missing. A clearer link can be provided by nascent‐chain dependent mRNA recruitment. The most well‐known example of translation coupling to targeting and translocation is the SRP pathway that is triggered by an SP exposed from the ribosome exit tunnel (Akopian et al., 2013). Many mitochondrial proteins also employ an N‐terminal targeting signal, but no SRP‐like pathway was identified for mitochondria so far. It is, however, clear that translation of the targeting signal and its binding to the TOM complex receptors are important to promote mRNA recruitment to the OMM (Eliyahu et al., 2010; Fazal et al., 2019; Gadir et al., 2011; Saint‐Georges et al., 2008). It is also clear that some matrix and inner membrane proteins can be selectively recruited for local translation while having similar N‐terminal targeting signals and topology as non‐recruited proteins (see Section 3.1). The mechanistic hypotheses explaining this selectivity fall into two categories, which are not mutually exclusive. The first category encompasses targeting factor hypotheses, while the second category includes hypotheses that suggest that there is no targeting factor, and the RNC recruitment is regulated by translation dynamics and nascent chain properties.

#### 4.2.1 Targeting factor hypothesis

An SRP‐like targeting factor was considered to explain co‐translational mRNA recruitment to the OMM. Such a factor should recognize an RNC that synthesizes a mitochondrial protein and promote the RNC docking to the OMM. The simplest recognition mechanism would act via binding a newly synthesized MTS exposed from the ribosome exit tunnel.

The factor that was proposed to act at the early steps of mitochondrial protein targeting was NAC (George et al., 1998). The deletion of yeast NAC leads to the decrease of the levels of mitochondrial proteins such as Fum1 and Mmf1 (George et al., 2002) and increased abundance of non‐imported precursors (Chen et al., 2023). In yeast, NAC was also shown to bind the OMM via the interactions with Om14 protein and promote mitochondria protein import in vitro (Lesnik et al., 2014). The structural basis of the NAC–Om14 interaction is unknown. The analysis of RNCs associated with different yeast NAC subunits showed that one of the beta‐NAC isoforms, Btt1, indeed prefers RNCs that translate mitochondrial proteins (del Alamo et al., 2011). The recent SeRP studies in yeast and worms showed that NAC does not specifically bind MTSs and that its binding to mitochondrial protein nascent chains follows the same pattern as that observed for cytonuclear proteins (Lee et al., 2026; Santos et al., 2026). In these nascent chains, NAC tends to interact with some of the unfolded domains and transmembrane helices (Lee et al., 2026; Santos et al., 2026). Depletion of NAC in human cells does not affect mRNA association with the TOM complex in a translation‐dependent manner, further supporting the idea that NAC does not act specifically on mitochondrial proteins (Zhu et al., 2025).

The effect of NAC depletion on mitochondrial protein import can be explained by three non‐specific mechanisms. First, NAC, as a conserved general chaperone, can help to bind hydrophobic protein stretches in preserving the import‐competence of mitochondrial precursors, especially the transmembrane ones (Lee et al., 2026; Santos et al., 2026). Second, studies in worms showed that NAC can displace the SRP from the nascent chains that do not have proper SPs and prevent mitochondrial protein mistargeting to the ER (Gamerdinger et al., 2015, 2019; Pech et al., 2010; Wiedmann & Prehn, 1999). Third, as a regulator of co‐translational modifications of the nascent chain, NAC can promote N‐terminal methionine cleavage and chain acetylation, which in turn can also be important for efficient targeting of some proteins (Nashed et al., 2023). The initially proposed role of NAC as a coordinator of MTS‐specific chaperone or targeting factor binding also cannot be ruled out (George et al., 1998).

The potential role of NAC in mitochondrial protein targeting raised similar questions regarding the other co‐translational chaperones. Groundbreaking SeRP studies of these chaperones in yeast also addressed their role in mitochondrial protein import. The deletion of Ssb led to aggregation of nascent proteins that were destined for the cytosol and organelles, including the mitochondria (Willmund et al., 2013). The co‐translational binding analysis of Ssb and TRiC did not reveal any specific recruitment pattern for mitochondrial proteins (Döring et al., 2017; Stein et al., 2019; Willmund et al., 2013). There was also no difference in Ssb binding between mitochondrial proteins translated next to the OMM and in the cytosol (Döring et al., 2017; Williams et al., 2014). In agreement with this, the in vivo pulse‐chase assay did not show a decreased import rate of proteins Lpd1 and Pim1 that are co‐translationally targeted based on SeRP data (Döring et al., 2017; Williams et al., 2014). Thus, there is so far no evidence that NAC, RAC, or TRiC are involved in co‐translational targeting, and only a few studies were done in organisms other than yeast. The co‐translational chaperones still may play a coordinating role. We know very little about their combined activities and interactions with post‐translational chaperones and with each other. Such complex cooperation might be important to select co‐translationally targeted proteins.

#### 4.2.2 Translation dynamics hypothesis

The targeting factor hypothesis implies a mechanistic link between translation and translocation. It also suggests that the targeting factor can somehow discriminate an MTS of a “co‐translational” and a “post‐translational” protein. An alternative hypothesis is that there is no targeting factor, and translocation simply starts once a newly synthesized targeting signal has a chance to interact with the TOM complex. A consequence of this model is that RNCs that expose a targeting signal in the nascent chain for a longer time have a higher chance to get recruited to the OMM before translation finishes (Arceo et al., 2022). Another interesting consequence is that the properties of the mature protein after MTS (here we call it coding sequence, CDS) are as important as the MTS to define the targeting mode. A simple prediction of such a model—that longer proteins are more likely to be co‐translationally targeted—agrees with high‐throughput measurements (Luo et al., 2025; Sylvestre et al., 2003; Williams et al., 2014; Zhu et al., 2025). SeRP studies in yeast and humans report that proteins shorter than 200 residues are rarely co‐translationally targeted unless they are RBP substrates (Luo et al., 2025; Williams et al., 2014). The massive OMM recruitment of yeast mRNAs encoding long MTS‐containing proteins like Atp1 and Atp3 upon CHX‐induced translation arrest also agrees with this model, as the MTS‐containing nascent chains have more time to interact with the TOM complex (Williams et al., 2014). Live imaging of individual yeast mRNAs with the MS2L system also showed that *ATP1* and *ATP3* mRNAs increase their association with the mitochondria after CHX treatment (Tsuboi et al., 2020). Thus, a gene property such as mRNA translation rate emerges as a possible regulator of co‐translational targeting (Arceo et al., 2022).

How translation rate can affect individual mRNA recruitment to mitochondria was investigated with yeast Tim50 protein as an example. Tim50, a component of the TIM23 complex residing in the IMM, contains a seven‐proline repeat in position 191–198 out of total 476. Such polyproline stretches cause ribosome stalling due to the difficulties of proline–proline peptide bond formation (Gutierrez et al., 2013). Removing the stretch from *TIM50* mRNA abolishes its localization to mitochondria, while inserting the stretch into another mRNA, like *ATP3*, leads to its association with mitochondria (Tsuboi et al., 2020). Similar proline repeats are also found in a few other yeast proteins like Tim54, Yta12, and Mss52, all of which are locally translated (Barba‐Aliaga et al., 2024; Williams et al., 2014), but there are only a few such obvious cases. Other features that might slow down translation, such as rare codon combinations or polybasic stretches, were not studied in the context of mitochondrial protein import and their individual impact on co‐translational targeting is not understood (Aguilar Rangel et al., 2024; Schuller & Green, 2018).

Can translation rate be a general mechanism to define whether a protein is targeted co‐translationally? Indeed, translation pauses were suggested to occur in strategic positions to promote protein folding and targeting (Pechmann et al., 2014; Pechmann & Frydman, 2013; Schuller & Green, 2018). In mice, footprinting of mRNA fragments trapped in disomes that indicate stalling positions showed that translation pauses are often found after ER and mitochondrial targeting sequences (Zhao et al., 2021). Datasets derived from ribosome profiling experiments allow us to estimate translation rates of each mRNA. A mathematical model of yeast translation based on this data showed that mRNAs that are constitutively found next to the OMM by SeRP indeed have slower translation rates than the other mRNAs (Arceo et al., 2022). This difference in translation rate, however, could not fully explain the SeRP data that show that a relatively low mRNA fraction is OMM‐associated (Williams et al., 2014). The model predicted much higher mRNA association to the OMM because most of the modeled RNCs would have at least one MTS able to bind the TOM complex. To reconcile with the SeRP data, the model had to incorporate an additional parameter, MTS maturation time (Arceo et al., 2022). The parameter introduces a hypothesis that the MTS is not active immediately after emerging from the exit tunnel but is temporarily kept inactive. The hypothesis suggests that there might be a specific molecular mechanism to inactivate the MTS.

The MTS inactivation hypothesis has support from structure–function studies in human cells. It was found that exchanging a region of 250 residues long that follows the MTS can turn post‐translationally targeted protein SUPV3L1 into a co‐translational protein, and a co‐translational protein ALDH18A1 into post‐translational (Luo et al., 2025). The authors suggested that a nascent chain feature directly after the SUPV3L1 MTS suppresses MTS activity. Exchanging the region following the SUPV3L1 MTS with an artificial soluble flexible linker XTEN also made SUPV3L1 co‐translationally targeted, favoring the idea that the MTS is sufficient for co‐translational targeting and its action depends on the context (Luo et al., 2025). Another study in mammalian cells found that the emergence of certain domains from the exit tunnel could trigger co‐translational targeting (Zhu et al., 2025). Importantly, simply extending the coding sequence was not enough for co‐translational targeting, suggesting that MTS activity required to initiate it does not depend purely on timing but on additional signals in the CDS. Thus, experimental findings that highlight the role of the nascent chain properties in MTS recognition add an additional level of complexity to the hypothesis of MTS maturation.

The targeting factor hypothesis based on the SRP mechanism suggests the active role of the factor that promotes targeting of the RNC to the organelle membrane. The hypothesis based on translation rate and MTS maturation suggests an opposite mechanism: the MTS and the TOM complex are sufficient for targeting, but RNC targeting is suppressed for most proteins until translation finishes. For a selected subset of RNCs, the suppression is relieved to allow co‐translational targeting. Such a relief signal can be a translation pause or a domain with a challenging folding pathway (Tsuboi et al., 2020; Zhu et al., 2025). How such a mechanism can be realized on a molecular level remains to be determined. This can involve co‐chaperones that bind the MTS (Juszkiewicz et al., 2025; Rödl et al., 2025). Their activity can be coordinated with co‐translational chaperones such as RAC, NAC, and TRiC that can recognize emerging protein domains, or with mRNA stability regulators such as the CCR4‐NOT complex (Chen et al., 2023).

### 4.3 Ribosome binding and nascent chain handover

Co‐translational protein import in the SRP pathway is mediated by the ribosome‐Sec61 complex (Voorhees et al., 2014). It allows the nascent chain to be translocated directly to the ER lumen from the ribosome exit tunnel. In mitochondrial import, the relationship between the ribosome and the TOM complex is most likely different and still poorly studied. The two main directions of investigation were to characterize the binding of ribosomes to the OMM and to determine if the nascent chain can be directly handed over during translation.

Electron microscopy of whole yeast cells and isolated mitochondria showed that ribosomes can bind the OMM or are found in close proximity to it (Chang et al., 2025; Gold et al., 2014, 2017; Kellems et al., 1974). A recent study that used in situ cryo‐electron tomography (cryo‐ET) reported the distributions and, for the first time, orientations of OMM‐associated ribosomes in the native cellular environment (Chang et al., 2025). The OMM‐associated ribosomes are not as abundant as ER‐associated ones, but many of them are oriented with their exit tunnel towards the membrane, suggesting participation in the protein translocation. When stabilized with CHX, ribosomes co‐purified together with yeast mitochondria oppose protein translocation sites (Gold et al., 2014). In their cellular environment, ribosomes tend to bind OMM regions that are closer to the IM with and without CHX treatment, indicating that these are also translocation sites (Chang et al., 2025). The ribosomes tend to occur in clusters, leaving some OMM patches bare; however, it is not clear how this positioning is determined. The role of cristae in co‐translational import site positioning was not supported by in situ cryo‐ET data (Chang et al., 2025; Gold et al., 2017).

The OMM‐associated ribosome structure determined by subtomogram averaging in budding yeast was similar between purified and intracellular mitochondrial samples (Chang et al., 2025; Gold et al., 2017). Both structures show the ribosome orientation similar to the Sec61‐bound ribosome, although the TOM complex is not visualized in the structure. Interestingly, unbiased template matching identified a slightly different orientation of OMM‐associated ribosomes in fission yeast (de Teresa‐Trueba et al., 2023). The resolution of the structures obtained by subtomogram averaging is not sufficient to clearly define the ribosomal regions that mediate the binding to the OMM. The absence of the TOM complex in the average suggests a flexible association. Weak ribosome binding is consistent with low yields of OMM‐associated ribosomes in the purified mitochondria fraction when the RNCs are not stabilized with CHX (Gold et al., 2017). ER‐bound ribosomes are easier to co‐purify with membranes. The interesting inconsistency between tomography and SeRP data lies in the response to CHX treatment. While SeRP shows dramatic recruitment of many mRNAs encoding MTS‐containing matrix proteins, in situ cryo‐ET only detects a modest 1.5‐time increase of the ribosomes positioned for import upon CHX treatment (Chang et al., 2025; Williams et al., 2014). These differences can be explained by weak and dynamic ribosome association with the OMM that allows turnover and change of the localized mRNA repertoire without significantly changing ribosome numbers. The only protein factor that was suggested to link ribosomes to the yeast OMM protein Om14 is NAC (Lesnik et al., 2014). The molecular details of this interaction and similar mechanisms in mammalian cells are not known. Thus, current data on ribosome binding to OMM is most consistent with the recruitment mechanism that relies on the MTS in the nascent chain and the TOM complex, without a structured ribosome‐translocon complex and a specific targeting mechanism.

How much time does the nascent chain linking the exit tunnel and the TOM complex spend in the cytosol and the molecular details of this handover are not known. The only protein that was studied is yeast fumarase (Fum1). The protein is only effectively translocated into purified yeast mitochondria when the import reaction is coupled to the in vitro translation reaction (Knox et al., 1998). In vivo, a Fum1 variant that had a TEV protease cleavage site engineered at the C‐terminus was not cleaved by cytosolic TEV (Yogev et al., 2007). Low accessibility of the precursor C‐terminus to the cytosolic protease cleavage suggests that the protein is quickly handed over from the ribosome to the TOM complex, but the exact timing is not known.

The import mode of Fum1 was suggested to be determined by its folding (Knox et al., 1998; Sass et al., 2003). If the folding occurs before translocation is completed, the folded domain can abort Fum1 import and induce retrotranslocation of the protein that already had its MTS cleaved off by matrix processing peptidase. Such an event generates the cytosolic form of Fum1 without MTS. When the import starts co‐translationally, the folding does not have time to prevent translocation, producing the matrix form of Fum1. This model has an interesting connection to the recent discovery that RNCs that are co‐purified with the human TOM complex have completed synthesis of certain protein domains (Zhu et al., 2025). It is not clear whether these precursor domains are bound outside of the purified TOM complex. The domain folding may also occur after translocation, on the matrix side, and help to anchor the RNC to the OMM. Another option is that the synthesized domain initiates MTS activation, helps to target the RNC to the OMM, and then the domain is unfolded before translocation. An artificial construct with two folded proteins connected by a linker was used to span TOM and TIM complexes to purify their supercomplex and determine its structure (Zhou et al., 2023). It remains to be determined if folding on the matrix side can happen during import of physiological substrates and can play a role in RNC binding to the OMM.

## 5 THE ROLE OF CO‐TRANSLATIONAL TARGETING AND TRANSLOCATION IN MITOCHONDRIAL BIOLOGY

The possible roles of local translation and co‐translational protein import are still not clearly understood. They can be split into two broad categories: adaptive roles in mitochondrial biogenesis and turnover, and potentially dangerous or unfavorable roles that require quality control.

### 5.1 Adaptive roles of co‐translational targeting

Local translation was proposed to play two main roles in mitochondrial function: adaptive regulation of mRNA expression by RBPs under changing conditions, and ensuring correct protein import and multiprotein complex assembly under normal conditions.

RBPs that bind mRNAs on the mitochondrial surface probably have mainly regulatory functions in unicellular and multicellular organisms (for review see Sharma & Fazal, 2024; Zilio et al., 2025). Yeast Puf3 can bind hundreds of mRNAs and either promote their degradation or translation (Lee & Tu, 2015; Miller et al., 2014; Olivas & Parker, 2000). The unified model of Puf3 function suggests that its activity depends on nutrient availability that determines phosphorylation status of Puf3 (Lee & Tu, 2015). In case of starvation, Puf3 can associate with mRNA granules and help safeguarding these mRNAs. Upon nutrient repletion, Puf3 can promote mRNA translation. Mammalian CLUH proteins are also linked to nutrient availability signaling because they are scaffolds for AMPK binding. AMPK responds to low ATP‐levels and is particularly important to adjust mitochondrial metabolism to survive energy deficiency (Herzig & Shaw, 2018). How exactly CLUH proteins transmit signals from AMPK into the changes in mitochondrial metabolism remains to be determined. Interestingly, mRNA translation efficiency can be altered based on the mRNA location, but the mechanism is yet unknown. Artificially anchoring an mRNA encoding GFP to the OMM was sufficient to increase expression levels of the GFP which did not have a targeting signal itself (Tsuboi et al., 2020). Thus, RBPs may not only have direct regulatory roles, but can also position mRNAs in a local environment that promotes or inhibits translation.

Neurons are particularly dependent on mitochondrial function due to their high energy demand. The large size of neurons makes RBP‐mediated mRNA transport and local translation particularly important to support oxidative phosphorylation in axons (De Pace et al., 2024; Liao et al., 2019). In this model, mitochondria that are far away from the cell body are supplied with new proteins by local translation. There are also specialized cases of local translation in neurons that do not directly support mitochondrial protein biogenesis but perform a regulatory function. PINK1 translation results in mitophagy and promotes mitochondrial quality control in axons (Harbauer et al., 2022), while local translation of MFF promotes mitochondrial fission and proper distribution of mtDNA among individual mitochondria (Fenton et al., 2024). Interestingly, *Pink1* mRNA tethering is regulated by AMPK. Phosphorylation of SYNJ2BP by AMPK promotes binding of SYNJ2 and *Pink1* mRNA. AMPK inhibition by insulin signaling leads to dephosphorylation of SYNJ2BP, release of *Pink1* mRNA, and reduced mitophagy (Hees, Wanderoy, et al., 2024). To sum up, RBPs can sense nutrient and energy status of the cell and influence mitochondrial biogenesis via general translational control or by affecting mitochondrial transport and turnover via regulation of specific transcripts.

Local translation and co‐translational protein import are considered directly useful for normal mitochondrial protein import, which is an essential process. Non‐imported precursor proteins are unfolded and can expose hydrophobic regions that promote aggregation and threaten cytosolic proteostasis (Boos et al., 2019; Krämer et al., 2023; Wrobel et al., 2015). A SeRP study in yeast found that under normal conditions inner membrane proteins are predominantly translated next to the OMM (Williams et al., 2014). Thus, co‐translational targeting may guide dangerous precursors directly to their destination and minimize precursor time in the cytosol, similarly to the SRP pathway. Indeed, exchanging the 3′UTR of yeast *ATP2* mRNA that is required for its localization to mitochondria with a heterologous UTR can impair respiration (Margeot et al., 2002). Whether this occurs due to an Atp2 protein import defect is unknown. Artificial mistargeting of mRNAs using MS2L and CP anchored to another organelle is a promising tool to investigate this question. Anchoring of mRNAs was used to show that localized translation is also important for yeast peroxisomes but was never systematically applied to mitochondrial‐protein encoding mRNAs (Dahan et al., 2022). Another way to alter mRNA localization is to change the features of the CDS. The removal of the polyproline repeat that is responsible for yeast *TIM50* mRNA recruitment to the OMM does not affect cell viability (Barba‐Aliaga et al., 2024; Tsuboi et al., 2020). It remains to be determined what is the adaptive advantage of locally translating Tim50.

Another challenge of mitochondrial biogenesis is the coordination of electron transport chain complexes assembly from the proteins encoded in two different genomes, nuclear and mitochondrial. It was suggested that preferential local translation of the complex subunits that share evolutionary origin can help to coordinate complex assembly across the two membranes (Garcia et al., 2006; Saint‐Georges et al., 2008; Sylvestre et al., 2003). There is a general program to synchronize cytosolic and mitochondrial translation but how the individual complex stoichiometry is maintained is less understood (Couvillion et al., 2016). The initial steps of mtDNA‐encoded protein biogenesis probably occur on the IMM region which is in close proximity to the OMM (Horten et al., 2024; Waltz et al., 2025). It would be interesting to uncover how translation on the matrix and cytosolic sides can be spatially coordinated.

### 5.2 The dangers of co‐translational import

Post‐translational mitochondrial protein import is the predominant pathway (Neupert, 1997). It can be beneficial, especially for rapidly growing organisms. In budding yeast, there are only around 10^4^ TOM complexes. During a cell replication cycle, these TOMs need to conduct proteins whose total length is 5 × 10^9^ amino acids (Morgenstern et al., 2017). Doing this entirely co‐translationally with the rate of 2–4 amino acids per second (Yan et al., 2016) would take much longer than the actual yeast cell cycle of 90 min. From this perspective, N‐terminal targeting signals present a challenge for fast‐growing cells because they can initiate translocation before translation is finished and enforce slow co‐translational import. This idea is consistent with the hypothesis that there is a mechanism that delays MTS activation for some time after its synthesis or regulates this delay based on the features of the nascent chain that follows the MTS (Arceo et al., 2022; Luo et al., 2025; Zhu et al., 2025). In this way, a few selected RNCs with activated MTSs would be permitted to bind the TOM complex and initiate co‐translational import, while the majority of RNCs making mitochondrial proteins would be prevented from binding the mitochondria, ensuring post‐translational import. It remains to be determined whether the MTS delay mechanism exists and how its disruption could affect cell growth.

The proteins that are recruited for co‐translational import can also create problems. One of the recruitment mechanisms is ribosome stalling, demonstrated by Tim50 protein that has a polyproline stretch. This stretch can be translated with the help of eIF5A factor (Caballero et al., 2025). Depletion of eIF5A in yeast (Hyp2) causes the drop in Tim50 levels and reduced mitochondrial import (Barba‐Aliaga et al., 2024). In macrophages, the loss of eIF5A also results in mitochondrial dysfunction (Puleston et al., 2019). Thus, programmed translation pauses need constant surveillance and correction by quality control systems.

The discrimination between productive programmed pauses and detrimental ribosome stalls depends on the balance between different stress pathways like p38/JNK or integrated stress response, and rescue mechanisms like eIF5A and RQC (Nanjaraj Urs et al., 2024; Wu et al., 2020). The balance can tip in different environmental and stress conditions, making translation pauses harmful and RQC essential for survival. One protein in this pathway, Vms1, was found to be particularly important for mitochondrial function in yeast. Vms1 (ANKZF1 in humans) is a tRNA hydrolase that releases nascent chains from 60S subunits generated by the disassembly of stalled ribosomes (Kuroha et al., 2018; Verma et al., 2018; Zurita Rendón et al., 2018). Before release, stuck nascent chains are elongated with alanines and threonines (CAT‐tailing) that help extracting and degrading them. However, these CAT‐tailed protein fragments cannot be efficiently degraded inside mitochondria, thus making aberrant products with N‐terminal MTS toxic (Izawa et al., 2017). Vms1 counteracts CAT‐tail formation and releases the nascent chains to the matrix where they can be degraded before CAT‐tails are synthesized (Bertram et al., 2025; Izawa et al., 2017). The physiological mitochondrial‐targeted substrates creating the problem that is resolved by Vms1 are not identified, but these may be co‐translationally imported proteins. This idea is supported by the finding that overexpression of mitochondrial proteins Shm1 and Leu9 with stop codon deletion that induces ribosomal stalling is lethal in a yeast strain lacking non‐stop decay factor Ski7 and ribosome splitting factor Dom34 (Izawa et al., 2012). According to SeRP data, Smh1 and Leu9 are both co‐translationally targeted, suggesting that this group of proteins might be potential troublemakers for mitochondrial homeostasis in case of translation stress (Izawa et al., 2012, 2017; Williams et al., 2014).

To sum up, while active targeting of hydrophobic proteins might be beneficial for cellular proteostasis, limiting the number of co‐translationally imported proteins and controlling their translation status at the TOM complex might also be necessary to promote cell growth and safeguard mitochondrial metabolism.

## 6 CONCLUSION

The advances in imaging and ribosome profiling helped to confirm that there are mRNAs that are preferentially translated next to the mitochondrial surface. Such recruitment can happen via RNA‐ and translation‐based mechanisms. Despite conceptual similarity with the SRP‐pathway in the ER, translation‐based recruitment to mitochondria probably has different mechanisms. A stable ribosome‐translocon complex was not found for the TOM complex, unlike for Sec61. RNC recruitment to the OMM usually depends on the targeting sequence and requires the TOM complex receptors. Several factors, such as NAC, that bind nascent chains in the cytosol were suggested to act as co‐translational targeting factors for mitochondrial proteins, but the mechanistic details of their involvement are unclear. A recent advance in the understanding of co‐translational targeting to mitochondria is the discovery that the protein sequence beyond the MTS may regulate co‐translational targeting either by its translation or folding dynamics. The discovery that RNC recruitment to mitochondria happens relatively late and depends on translation rate suggests that co‐translational targeting is not a separate pathway but is deeply integrated with cytosolic protein maturation. It is becoming clear that co‐translational targeting, as translation itself, is highly dynamic and can be regulated depending on the growth conditions, making it a difficult subject to study.

## 7 OPEN QUESTIONS AND OUTLOOK

We are optimistic that the progress in imaging and sequencing techniques that allows us to study local translation at smaller distance and time scales will help to tackle some of the open questions in the field. It remains to be determined how the nascent chain properties determine RNC recruitment to the OMM. It would be interesting to find out how different co‐translational chaperones recognize the properties of the nascent chain and how the co‐translational targeting process is connected to co‐translational folding and complex assembly, as they may share the molecular machinery. Another important outstanding question is the relation between translation and translocation dynamics. Does translocation start immediately after RNC binding to the OMM? It would be interesting to map the relative positions of mRNAs, RNCs, and TOM complexes on the OMM surface using high‐resolution imaging or spatial proteomics techniques. The methods that allow changing mRNA localization can help to answer another major question: what is the biological role of local translation and co‐translational import? Do these processes primarily regulate gene expression, or ensure efficient protein translocation? Another new hypothesis to test is that co‐translational import into mitochondria might be negatively regulated to ensure efficient post‐translational import. Finally, investigating local translation and co‐translational import in different organisms and cell types will help to answer the question of how universal this process is and how it supports different physiological functions.

## AUTHOR CONTRIBUTIONS

**Yury S. Bykov:** Writing – original draft; writing – review and editing. **Nikita A. Kvasov:** Writing – original draft; writing – review and editing.

## CONFLICT OF INTEREST STATEMENT

The authors declare no conflicts of interest.

## Data Availability

Data sharing not applicable to this article as no datasets were generated or analysed during the current study.

## References

[pro70682-bib-0001] Ades IZ , Butow RA . The transport of proteins into yeast mitochondria. Kinetics and pools. J Biol Chem. 1980a;255:9925–9935. 6448842

[pro70682-bib-0002] Ades IZ , Butow RA . The products of mitochondria‐bound cytoplasmic polysomes in yeast. J Biol Chem. 1980b;255:9918–9924. 6448841

[pro70682-bib-0003] Aguilar Rangel M , Stein K , Frydman J . A machine learning approach uncovers principles and determinants of eukaryotic ribosome pausing. Sci Adv. 2024;10:eado0738. 39423268 10.1126/sciadv.ado0738 PMC11488575

[pro70682-bib-0004] Akopian D , Shen K , Zhang X , Shan S . Signal recognition particle: an essential protein‐targeting machine. Annu Rev Biochem. 2013;82:693–721. 23414305 10.1146/annurev-biochem-072711-164732 PMC3805129

[pro70682-bib-0005] Akram S , Zittlau KI , Sharma K , Fitzgerald JC , Rafiq N , Maček B , et al. Proximity labeling reveals RNA‐binding proteins associating with the human mitochondrial import receptor TOMM20. J Proteome Res. 2026;25:1055–1070. 41427806 10.1021/acs.jproteome.5c00905 PMC12888002

[pro70682-bib-0006] Araiso Y , Tsutsumi A , Qiu J , Imai K , Shiota T , Song J , et al. Structure of the mitochondrial import gate reveals distinct preprotein paths. Nature. 2019;575:395–401. 31600774 10.1038/s41586-019-1680-7

[pro70682-bib-0007] Arceo XG , Koslover EF , Zid BM , Brown AI . Mitochondrial mRNA localization is governed by translation kinetics and spatial transport. PLoS Comput Biol. 2022;18:e1010413. 35984860 10.1371/journal.pcbi.1010413 PMC9432724

[pro70682-bib-0008] Ast T , Cohen G , Schuldiner M . A network of cytosolic factors targets SRP‐independent proteins to the endoplasmic reticulum. Cell. 2013;152:1134–1145. 23452858 10.1016/j.cell.2013.02.003

[pro70682-bib-0009] Aviram N , Ast T , Costa EA , Arakel EC , Chuartzman SG , Jan CH , et al. The SND proteins constitute an alternative targeting route to the endoplasmic reticulum. Nature. 2016;540:134–138. 27905431 10.1038/nature20169 PMC5513701

[pro70682-bib-0010] Backes S , Bykov YS , Flohr T , Räschle M , Zhou J , Lenhard S , et al. The chaperone‐binding activity of the mitochondrial surface receptor Tom70 protects the cytosol against mitoprotein‐induced stress. Cell Rep. 2021;35:108936. 33826901 10.1016/j.celrep.2021.108936 PMC7615001

[pro70682-bib-0011] Backes S , Hess S , Boos F , Woellhaf MW , Gödel S , Jung M , et al. Tom70 enhances mitochondrial preprotein import efficiency by binding to internal targeting sequences. J Cell Biol. 2018;217:1369–1382. 29382700 10.1083/jcb.201708044 PMC5881500

[pro70682-bib-0012] Balchin D , Hayer‐Hartl M , Hartl FU . In vivo aspects of protein folding and quality control. Science. 2016;353:aac4354. 27365453 10.1126/science.aac4354

[pro70682-bib-0013] Barba‐Aliaga M , Bernal V , Rong C , Volfbeyn ME , Zhang K , Zid BM , et al. eIF5A controls mitoprotein import by relieving ribosome stalling at TIM50 translocase mRNA. J Cell Biol. 2024;223:e202404094. 39509053 10.1083/jcb.202404094 PMC11551009

[pro70682-bib-0014] Beckett D , Kovaleva E , Schatz PJ . A minimal peptide substrate in biotin holoenzyme synthetase‐catalyzed biotinylation. Protein Sci. 1999;8:921–929. 10211839 10.1110/ps.8.4.921 PMC2144313

[pro70682-bib-0015] Bertolini M , Fenzl K , Kats I , Wruck F , Tippmann F , Schmitt J , et al. Interactions between nascent proteins translated by adjacent ribosomes drive homomer assembly. Science. 2021;371:57–64. 33384371 10.1126/science.abc7151 PMC7613021

[pro70682-bib-0016] Bertram N , Izawa T , Thoma F , Schwenkert S , Duvezin‐Caubet S , Park S‐H , et al. Delayed protein translocation protects mitochondria against toxic CAT‐tailed proteins. Mol Cell. 2025;85:4082–4092.e7. 41118763 10.1016/j.molcel.2025.09.030

[pro70682-bib-0017] Boos F , Krämer L , Groh C , Jung F , Haberkant P , Stein F , et al. Mitochondrial protein‐induced stress triggers a global adaptive transcriptional programme. Nat Cell Biol. 2019;21:442–451. 30886345 10.1038/s41556-019-0294-5

[pro70682-bib-0018] Bourke AM , Schwarz A , Schuman EM . De‐centralizing the central dogma: mRNA translation in space and time. Mol Cell. 2023;83:452–468. 36669490 10.1016/j.molcel.2022.12.030

[pro70682-bib-0019] Brito Querido J , Díaz‐López I , Ramakrishnan V . The molecular basis of translation initiation and its regulation in eukaryotes. Nat Rev Mol Cell Biol. 2024;25:168–186. 38052923 10.1038/s41580-023-00624-9

[pro70682-bib-0020] Brix J , Dietmeier K , Pfanner N . Differential recognition of preproteins by the purified cytosolic domains of the mitochondrial import receptors Tom20, Tom22, and Tom70. J Biol Chem. 1997;272:20730–20735. 9252394 10.1074/jbc.272.33.20730

[pro70682-bib-0021] Bulmash AS , Fischer AD , Russo J , Mueller SM , Olivas WM . Yeast Puf3p‐mediated mRNA decay is regulated by carbon source‐specific differential interaction of Puf3p with Pop2p and Yak1p. FEBS Lett. 2023;597:1606–1622. 37060252 10.1002/1873-3468.14624

[pro70682-bib-0022] Bykov YS , Rapaport D , Herrmann JM , Schuldiner M . Cytosolic events in the biogenesis of mitochondrial proteins. Trends Biochem Sci. 2020;45:650–667. 32409196 10.1016/j.tibs.2020.04.001

[pro70682-bib-0023] Caballero D , Sutter BM , Xing Z , Wang C , Choo E , Wang Y , et al. The yeast Mkt1/Pbp1 complex promotes adaptive responses to respiratory growth. J Cell Biol. 2025;224:e202411169. 40801888 10.1083/jcb.202411169 PMC12345631

[pro70682-bib-0024] Chang Y‐T , Barad BA , Hamid J , Rahmani H , Zid BM , Grotjahn DA . Cytoplasmic ribosomes on mitochondria alter the local membrane environment for protein import. J Cell Biol. 2025;224:e202407110. 40047641 10.1083/jcb.202407110 PMC11893167

[pro70682-bib-0025] Chartron JW , Hunt KCL , Frydman J . Cotranslational signal‐independent SRP preloading during membrane targeting. Nature. 2016;536:224–228. 27487213 10.1038/nature19309 PMC5120976

[pro70682-bib-0026] Chen S , Allen G , Panasenko OO , Collart MA . Not4‐dependent targeting of MMF1 mRNA to mitochondria limits its expression via ribosome pausing, Egd1 ubiquitination, Caf130, no‐go‐decay and autophagy. Nucleic Acids Res. 2023;51:5022–5039. 37094076 10.1093/nar/gkad299 PMC10250226

[pro70682-bib-0027] Collart MA , Weiss B . Ribosome pausing, a dangerous necessity for co‐translational events. Nucleic Acids Res. 2020;48:1043–1055. 31598688 10.1093/nar/gkz763 PMC7026645

[pro70682-bib-0028] Couvillion MT , Soto IC , Shipkovenska G , Churchman LS . Synchronized mitochondrial and cytosolic translation programs. Nature. 2016;533:499–503. 27225121 10.1038/nature18015 PMC4964289

[pro70682-bib-0029] Cox RT , Spradling AC . Clueless, a conserved Drosophila gene required for mitochondrial subcellular localization, interacts genetically with parkin. Dis Model Mech. 2009;2:490–499. 19638420 10.1242/dmm.002378 PMC2737057

[pro70682-bib-0030] Dahan N , Bykov YS , Boydston EA , Fadel A , Gazi Z , Hochberg‐Laufer H , et al. Peroxisome function relies on organelle‐associated mRNA translation. Sci Adv. 2022;8:eabk2141. 35020435 10.1126/sciadv.abk2141 PMC8754406

[pro70682-bib-0031] De Pace R , Ghosh S , Ryan VH , Sohn M , Jarnik M , Rezvan Sangsari P , et al. Messenger RNA transport on lysosomal vesicles maintains axonal mitochondrial homeostasis and prevents axonal degeneration. Nat Neurosci. 2024;27:1087–1102. 38600167 10.1038/s41593-024-01619-1 PMC11156585

[pro70682-bib-0032] de Teresa‐Trueba I , Goetz SK , Mattausch A , Stojanovska F , Zimmerli CE , Toro‐Nahuelpan M , et al. Convolutional networks for supervised mining of molecular patterns within cellular context. Nat Methods. 2023;20:284–294. 36690741 10.1038/s41592-022-01746-2 PMC9911354

[pro70682-bib-0033] Dekker PJT , Ryan MT , Brix J , Müller H , Hönlinger A , Pfanner N . Preprotein translocase of the outer mitochondrial membrane: molecular dissection and assembly of the general import pore complex. Mol Cell Biol. 1998;18:6515–6524. 9774667 10.1128/mcb.18.11.6515 PMC109237

[pro70682-bib-0034] del Alamo M , Hogan DJ , Pechmann S , Albanese V , Brown PO , Frydman J . Defining the specificity of cotranslationally acting chaperones by systematic analysis of mRNAs associated with ribosome‐nascent chain complexes. PLoS Biol. 2011;9:e1001100. 21765803 10.1371/journal.pbio.1001100 PMC3134442

[pro70682-bib-0035] Döring K , Ahmed N , Riemer T , Suresh HG , Vainshtein Y , Habich M , et al. Profiling Ssb‐nascent chain interactions reveals principles of Hsp70‐assisted folding. Cell. 2017;170:298–311.e20. 28708998 10.1016/j.cell.2017.06.038 PMC7343536

[pro70682-bib-0036] Eilers M , Schatz G . Binding of a specific ligand inhibits import of a purified precursor protein into mitochondria. Nature. 1986;322:228–232. 3016548 10.1038/322228a0

[pro70682-bib-0037] Eliyahu E , Pnueli L , Melamed D , Scherrer T , Gerber AP , Pines O , et al. Tom20 mediates localization of mRNAs to mitochondria in a translation‐dependent manner. Mol Cell Biol. 2010;30:284–294. 19858288 10.1128/MCB.00651-09 PMC2798288

[pro70682-bib-0038] Fazal FM , Han S , Parker KR , Kaewsapsak P , Xu J , Boettiger AN , et al. Atlas of subcellular RNA localization revealed by APEX‐Seq. Cell. 2019;178:473–490.e26. 31230715 10.1016/j.cell.2019.05.027 PMC6786773

[pro70682-bib-0039] Fenton AR , Peng R , Bond C , Hugelier S , Lakadamyali M , Chang Y‐W , et al. FMRP regulates MFF translation to locally direct mitochondrial fission in neurons. Nat Cell Biol. 2024;26:2061–2074. 39548330 10.1038/s41556-024-01544-2 PMC11628401

[pro70682-bib-0040] Fields SD , Conrad MN , Clarke M . The S. cerevisiae CLU1 and D. discoideum cluA genes are functional homologues that influence mitochondrial morphology and distribution. J Cell Sci. 1998;111:1717–1727. 9601101 10.1242/jcs.111.12.1717

[pro70682-bib-0041] Gabrovsek L , Collins KB , Aggarwal S , Saunders LM , Lau H‐T , Suh D , et al. A‐kinase‐anchoring protein 1 (dAKAP1)‐based signaling complexes coordinate local protein synthesis at the mitochondrial surface. J Biol Chem. 2020;295:10749–10765. 32482893 10.1074/jbc.RA120.013454 PMC7397098

[pro70682-bib-0042] Gadir N , Haim‐Vilmovsky L , Kraut‐Cohen J , Gerst JE . Localization of mRNAs coding for mitochondrial proteins in the yeast Saccharomyces cerevisiae. RNA. 2011;17:1551–1565. 21705432 10.1261/rna.2621111 PMC3153978

[pro70682-bib-0043] Gamerdinger M , Deuerling E . Cotranslational sorting and processing of newly synthesized proteins in eukaryotes. Trends Biochem Sci. 2024;49:105–118. 37919225 10.1016/j.tibs.2023.10.003

[pro70682-bib-0044] Gamerdinger M , Hanebuth MA , Frickey T , Deuerling E . The principle of antagonism ensures protein targeting specificity at the endoplasmic reticulum. Science. 2015;348:201–207. 25859040 10.1126/science.aaa5335

[pro70682-bib-0045] Gamerdinger M , Kobayashi K , Wallisch A , Kreft SG , Sailer C , Schlömer R , et al. Early scanning of nascent polypeptides inside the ribosomal tunnel by NAC. Mol Cell. 2019;75:996–1006.e8. 31377116 10.1016/j.molcel.2019.06.030

[pro70682-bib-0046] Gao J , Schatton D , Martinelli P , Hansen H , Pla‐Martin D , Barth E , et al. CLUH regulates mitochondrial biogenesis by binding mRNAs of nuclear‐encoded mitochondrial proteins. J Cell Biol. 2014;207:213–223. 25349259 10.1083/jcb.201403129 PMC4210445

[pro70682-bib-0047] Garcia M , Darzacq X , Delaveau T , Jourdren L , Singer RH , Jacq C . Mitochondria‐associated yeast mRNAs and the biogenesis of molecular complexes. Mol Biol Cell. 2006;18:362–368. 17108321 10.1091/mbc.E06-09-0827 PMC1783778

[pro70682-bib-0048] García‐Rodríguez LJ , Gay AC , Pon LA . Puf3p, a Pumilio family RNA binding protein, localizes to mitochondria and regulates mitochondrial biogenesis and motility in budding yeast. J Cell Biol. 2007;176:197–207. 17210948 10.1083/jcb.200606054 PMC2063939

[pro70682-bib-0049] Gautschi M , Lilie H , Fünfschilling U , Mun A , Ross S , Lithgow T , et al. RAC, a stable ribosome‐associated complex in yeast formed by the DnaK‐DnaJ homologs Ssz1p and zuotin. Proc Natl Acad Sci. 2001;98:3762–3767. 11274393 10.1073/pnas.071057198 PMC31126

[pro70682-bib-0050] George R , Beddoe T , Landl K , Lithgow T . The yeast nascent polypeptide‐associated complex initiates protein targeting to mitochondria in vivo. Proc Natl Acad Sci. 1998;95:2296–2301. 9482879 10.1073/pnas.95.5.2296 PMC19325

[pro70682-bib-0051] George R , Walsh P , Beddoe T , Lithgow T . The nascent polypeptide‐associated complex (NAC) promotes interaction of ribosomes with the mitochondrial surface in vivo. FEBS Lett. 2002;516:213–216. 11959135 10.1016/s0014-5793(02)02528-0

[pro70682-bib-0052] Gerber AP , Herschlag D , Brown PO . Extensive association of functionally and cytotopically related mRNAs with Puf family RNA‐binding proteins in yeast. PLoS Biol. 2004;2:e79. 15024427 10.1371/journal.pbio.0020079 PMC368173

[pro70682-bib-0053] Ginsberg MD , Feliciello A , Jones JK , Avvedimento EV , Gottesman ME . PKA‐dependent binding of mRNA to the mitochondrial AKAP121 protein. J Mol Biol. 2003;327:885–897. 12654270 10.1016/s0022-2836(03)00173-6

[pro70682-bib-0054] Gold VA , Chroscicki P , Bragoszewski P , Chacinska A . Visualization of cytosolic ribosomes on the surface of mitochondria by electron cryo‐tomography. EMBO Rep. 2017;18:1786–1800. 28827470 10.15252/embr.201744261 PMC5623831

[pro70682-bib-0055] Gold VAM , Ieva R , Walter A , Pfanner N , van der Laan M , Kühlbrandt W . Visualizing active membrane protein complexes by electron cryotomography. Nat Commun. 2014;5:4129. 24942077 10.1038/ncomms5129 PMC4090714

[pro70682-bib-0056] Gutierrez E , Shin B‐S , Woolstenhulme CJ , Kim J‐R , Saini P , Buskirk AR , et al. eIF5A promotes translation of polyproline motifs. Mol Cell. 2013;51:35–45. 23727016 10.1016/j.molcel.2013.04.021 PMC3744875

[pro70682-bib-0057] Harbauer AB , Hees JT , Wanderoy S , Segura I , Gibbs W , Cheng Y , et al. Neuronal mitochondria transport *Pink1* mRNA via synaptojanin 2 to support local mitophagy. Neuron. 2022;110:1516–1531.e9. 35216662 10.1016/j.neuron.2022.01.035 PMC9081165

[pro70682-bib-0058] Hayashi S , Iwamoto K , Yoshihisa T . Puf3p facilitates fermentative mitochondrial functions via monosome‐enriched nuclear‐encoded mitochondrial mRNAs in budding yeast. bioRxiv. 2022. 10.1101/2022.04.10.487782v1

[pro70682-bib-0059] Hees JT , Segura I , Schneider A , Schifferer M , Misgeld T , Harbauer AB . ER‐associated biogenesis of PINK1 preprotein for neuronal mitophagy. bioRxiv. 2024. 10.1101/2024.06.21.600039v1

[pro70682-bib-0060] Hees JT , Wanderoy S , Lindner J , Helms M , Murali Mahadevan H , Harbauer AB . Insulin signalling regulates Pink1 mRNA localization via modulation of AMPK activity to support PINK1 function in neurons. Nat Metab. 2024;6:514–530. 38504131 10.1038/s42255-024-01007-w PMC10963278

[pro70682-bib-0061] Herzig S , Shaw RJ . AMPK: guardian of metabolism and mitochondrial homeostasis. Nat Rev Mol Cell Biol. 2018;19:121–135. 28974774 10.1038/nrm.2017.95 PMC5780224

[pro70682-bib-0062] Hill K , Model K , Ryan MT , Dietmeier K , Martin F , Wagner R , et al. Tom40 forms the hydrophilic channel of the mitochondrial import pore for preproteins. Nature. 1998;395:516–521. 9774109 10.1038/26780

[pro70682-bib-0063] Horten P , Song K , Garlich J , Hardt R , Colina‐Tenorio L , Horvath SE , et al. Identification of MIMAS, a multifunctional mega‐assembly integrating metabolic and respiratory biogenesis factors of mitochondria. Cell Rep. 2024;43:113772. 38393949 10.1016/j.celrep.2024.113772 PMC11010658

[pro70682-bib-0064] Huang LJ , Durick K , Weiner JA , Chun J , Taylor SS . Identification of a novel protein kinase A anchoring protein that binds both type I and type II regulatory subunits. J Biol Chem. 1997;272:8057–8064. 9065479 10.1074/jbc.272.12.8057

[pro70682-bib-0065] Inada T , Beckmann R . Mechanisms of translation‐coupled quality control. J Mol Biol. 2024;436:168496. 38365086 10.1016/j.jmb.2024.168496

[pro70682-bib-0066] Ingolia NT , Hussmann JA , Weissman JS . Ribosome profiling: global views of translation. Cold Spring Harb Perspect Biol. 2019;11:a032698. 30037969 10.1101/cshperspect.a032698 PMC6496350

[pro70682-bib-0067] Izawa T , Park S‐H , Zhao L , Hartl FU , Neupert W . Cytosolic protein Vms1 links ribosome quality control to mitochondrial and cellular homeostasis. Cell. 2017;171:890–903.e18. 29107329 10.1016/j.cell.2017.10.002

[pro70682-bib-0068] Izawa T , Tsuboi T , Kuroha K , Inada T , Nishikawa S , Endo T . Roles of Dom34:Hbs1 in nonstop protein clearance from translocators for normal organelle protein influx. Cell Rep. 2012;2:447–453. 22981232 10.1016/j.celrep.2012.08.010

[pro70682-bib-0069] Jan CH , Williams CC , Weissman JS . Principles of ER cotranslational translocation revealed by proximity‐specific ribosome profiling. Science. 2014;346:1257521. 25378630 10.1126/science.1257521 PMC4285348

[pro70682-bib-0070] Juszkiewicz S , Peak‐Chew S‐Y , Hegde RS . Mechanism of chaperone recruitment and retention on mitochondrial precursors. Mol Biol Cell. 2025;36:ar39. 39878680 10.1091/mbc.E25-01-0035 PMC7617541

[pro70682-bib-0071] Kellems RE , Allison VF , Butow RA . Cytoplasmic type 80 S ribosomes associated with yeast mitochondria II. Evidence for the association of cytoplasmic ribosomes with the outer mitochondrial membrane in situ. J Biol Chem. 1974;249:3297–3303. 4598123

[pro70682-bib-0072] Kellems RE , Allison VF , Butow RA . Cytoplasmic type 80S ribosomes associated with yeast mitochondria. IV. Attachment of ribosomes to the outer membrane of isolated mitochondria. J Cell Biol. 1975;65:1–14. 1092698 10.1083/jcb.65.1.1 PMC2111154

[pro70682-bib-0073] Kellems RE , Butow RA . Cytoplasmic‐type 80 S ribosomes associated with yeast mitochondria I. Evidence for ribosome binding sites on yeast mitochondria. J Biol Chem. 1972;247:8043–8050. 4629740

[pro70682-bib-0074] Knox C , Sass E , Neupert W , Pines O . Import into mitochondria, folding and retrograde movement of fumarase in yeast. J Biol Chem. 1998;273:25587–25593. 9748223 10.1074/jbc.273.40.25587

[pro70682-bib-0076] Kramer G , Shiber A , Bukau B . Mechanisms of cotranslational maturation of newly synthesized proteins. Annu Rev Biochem. 2019;88:337–364. 30508494 10.1146/annurev-biochem-013118-111717

[pro70682-bib-0075] Krämer L , Dalheimer N , Räschle M , Storchová Z , Pielage J , Boos F , et al. MitoStores: chaperone‐controlled protein granules store mitochondrial precursors in the cytosol. EMBO J. 2023;42:e112309. 36704946 10.15252/embj.2022112309 PMC10068336

[pro70682-bib-0077] Kuroha K , Zinoviev A , Hellen CUT , Pestova TV . Release of ubiquitinated and non‐ubiquitinated nascent chains from stalled mammalian ribosomal complexes by ANKZF1 and Ptrh1. Mol Cell. 2018;72:286–302.e8. 30244831 10.1016/j.molcel.2018.08.022 PMC6344051

[pro70682-bib-0079] Lee C‐D , Tu BP . Glucose‐regulated phosphorylation of the PUF protein Puf3 regulates the translational fate of its bound mRNAs and association with RNA granules. Cell Rep. 2015;11:1638–1650. 26051939 10.1016/j.celrep.2015.05.014 PMC4472502

[pro70682-bib-0078] Lee JH , Rabl L , Gamerdinger M , Goyal V , Khakzar KM , Barbosa NM , et al. NAC controls nascent chain fate through tunnel sensing and chaperone action. Nature. 2026;652:230–239. 41430436 10.1038/s41586-025-10058-2 PMC13043293

[pro70682-bib-0080] Lesnik C , Cohen Y , Atir‐Lande A , Schuldiner M , Arava Y . OM14 is a mitochondrial receptor for cytosolic ribosomes that supports co‐translational import into mitochondria. Nat Commun. 2014;5:5711. 25487825 10.1038/ncomms6711 PMC4268710

[pro70682-bib-0081] Lewis BM , Cho CY , Her H‐L , Mizrahi O , Hunter T , Yeo GW . LARP4 is an RNA‐binding protein that binds nuclear‐encoded mitochondrial mRNAs to promote mitochondrial function. RNA. 2024;30:223–239. 38164626 10.1261/rna.079799.123 PMC10870378

[pro70682-bib-0082] Liao Y‐C , Fernandopulle MS , Wang G , Choi H , Hao L , Drerup CM , et al. RNA granules hitchhike on lysosomes for long‐distance transport, using annexin A11 as a molecular tether. Cell. 2019;179:147–164.e20. 31539493 10.1016/j.cell.2019.08.050 PMC6890474

[pro70682-bib-0083] Lin R‐Y , Moss SB , Rubin CS . Characterization of S‐AKAP84, a novel developmentally regulated a kinase anchor protein of male germ cells. J Biol Chem. 1995;270:27804–27811. 7499250 10.1074/jbc.270.46.27804

[pro70682-bib-0084] Luo J , Khandwala S , Hu J , Lee S‐Y , Hickey KL , Levine ZG , et al. Proximity‐specific ribosome profiling reveals the logic of localized mitochondrial translation. Cell. 2025;188:5589–5604.e17. 40876456 10.1016/j.cell.2025.08.002 PMC12650760

[pro70682-bib-0085] Mallik S , Venezian J , Lobov A , Heidenreich M , Garcia‐Seisdedos H , Yeates TO , et al. Structural determinants of co‐translational protein complex assembly. Cell. 2025;188:764–777.e22. 39708808 10.1016/j.cell.2024.11.013

[pro70682-bib-0086] Marc P , Margeot A , Devaux F , Blugeon C , Corral‐Debrinski M , Jacq C . Genome‐wide analysis of mRNAs targeted to yeast mitochondria. EMBO Rep. 2002;3:159–164. 11818335 10.1093/embo-reports/kvf025 PMC1083966

[pro70682-bib-0087] Margeot A , Blugeon C , Sylvestre J , Vialette S , Jacq C , Corral‐Debrinski M . In Saccharomyces cerevisiae, ATP2 mRNA sorting to the vicinity of mitochondria is essential for respiratory function. EMBO J. 2002;21:6893–6904. 12486010 10.1093/emboj/cdf690 PMC139110

[pro70682-bib-0088] Mi L , Khajouei S , You M . Multiplexed RNA imaging and in situ profiling in living cells. Chem Sci. 2025;16:21152–21173. 41210282 10.1039/d5sc06220a PMC12589969

[pro70682-bib-0089] Miller MA , Russo J , Fischer AD , Lopez Leban FA , Olivas WM . Carbon source‐dependent alteration of Puf3p activity mediates rapid changes in the stabilities of mRNAs involved in mitochondrial function. Nucleic Acids Res. 2014;42:3954–3970. 24371272 10.1093/nar/gkt1346 PMC3973295

[pro70682-bib-0090] Miller‐Fleming L , Au WH , Raik L , Rebelo‐Guiomar P , Schmitz J , Cho HY , et al. Clu1/Clu form mitochondria‐associated granules upon metabolic transitions and regulate mitochondrial protein translation via ribosome interactions. PLoS Genet. 2025;21:e1011773. 40623095 10.1371/journal.pgen.1011773 PMC12262889

[pro70682-bib-0091] Morgenstern M , Stiller SB , Lübbert P , Peikert CD , Dannenmaier S , Drepper F , et al. Definition of a high‐confidence mitochondrial proteome at quantitative scale. Cell Rep. 2017;19:2836–2852. 28658629 10.1016/j.celrep.2017.06.014 PMC5494306

[pro70682-bib-0092] Nanjaraj Urs AN , Lasehinde V , Kim L , McDonald E , Yan LL , Zaher HS . Inability to rescue stalled ribosomes results in overactivation of the integrated stress response. J Biol Chem. 2024;300:107290. 38636664 10.1016/j.jbc.2024.107290 PMC11106528

[pro70682-bib-0093] Nashed S , Barbry HE , Benchouaia M , Dijoux‐Maréchal A , Delaveau T , Ruiz‐Gutierrez N , et al. Functional mapping of N‐terminal residues in the yeast proteome uncovers novel determinants for mitochondrial protein import. PLoS Genet. 2023;19:e1010848. 37585488 10.1371/journal.pgen.1010848 PMC10482271

[pro70682-bib-0094] Nauerz C , Pines O , Herrmann JM . Biogenesis and function of the mitochondrial solute carrier (SLC25) family in yeast. Biol Chem. 2025;406:505–516. 40836422 10.1515/hsz-2025-0152 PMC12783890

[pro70682-bib-0095] Nemoto Y , De Camilli P . Recruitment of an alternatively spliced form of synaptojanin 2 to mitochondria by the interaction with the PDZ domain of a mitochondrial outer membrane protein. EMBO J. 1999;18:2991–3006. 10357812 10.1093/emboj/18.11.2991 PMC1171381

[pro70682-bib-0096] Neupert W . Protein import into mitochondria. Annu Rev Biochem. 1997;66:863–917. 9242927 10.1146/annurev.biochem.66.1.863

[pro70682-bib-0097] Nishanth MJ , Simon B . Functions, mechanisms and regulation of Pumilio/Puf family RNA binding proteins: a comprehensive review. Mol Biol Rep. 2020;47:785–807. 31643042 10.1007/s11033-019-05142-6

[pro70682-bib-0098] Olivas W , Parker R . The Puf3 protein is a transcript‐specific regulator of mRNA degradation in yeast. EMBO J. 2000;19:6602–6611. 11101532 10.1093/emboj/19.23.6602 PMC305854

[pro70682-bib-0099] Passmore LA , Coller J . Roles of mRNA poly(A) tails in regulation of eukaryotic gene expression. Nat Rev Mol Cell Biol. 2022;23:93–106. 34594027 10.1038/s41580-021-00417-y PMC7614307

[pro70682-bib-0100] Pech M , Spreter T , Beckmann R , Beatrix B . Dual binding mode of the nascent polypeptide‐associated complex reveals a novel universal adapter site on the ribosome. J Biol Chem. 2010;285:19679–19687. 20410297 10.1074/jbc.M109.092536 PMC2885246

[pro70682-bib-0101] Pechmann S , Chartron JW , Frydman J . Local slowdown of translation by nonoptimal codons promotes nascent‐chain recognition by SRP in vivo. Nat Struct Mol Biol. 2014;21:1100–1105. 25420103 10.1038/nsmb.2919 PMC4488850

[pro70682-bib-0102] Pechmann S , Frydman J . Evolutionary conservation of codon optimality reveals hidden signatures of cotranslational folding. Nat Struct Mol Biol. 2013;20:237–243. 23262490 10.1038/nsmb.2466 PMC3565066

[pro70682-bib-0103] Pichon X , Robert M‐C , Bertrand E , Singer RH , Tutucci E . New generations of MS2 variants and MCP fusions to detect single mRNAs in living eukaryotic cells. Methods Mol Biol (Clifton, NJ). 2020;2166:121–144. 10.1007/978-1-0716-0712-1_7 PMC8950302 32710406

[pro70682-bib-0104] Puleston DJ , Buck MD , Klein Geltink RI , Kyle RL , Caputa G , O'Sullivan D , et al. Polyamines and eIF5A hypusination modulate mitochondrial respiration and macrophage activation. Cell Metab. 2019;30:352–363.e8. 31130465 10.1016/j.cmet.2019.05.003 PMC6688828

[pro70682-bib-0105] Quenault T , Lithgow T , Traven A . PUF proteins: repression, activation and mRNA localization. Trends Cell Biol. 2011;21:104–112. 21115348 10.1016/j.tcb.2010.09.013

[pro70682-bib-0106] Rödl S , Hoffman Y , Jung F , Egeler A , Nutz A , Šimončík O , et al. A protein‐specific priority code in presequences determines the efficiency of mitochondrial protein import. PLoS Biol. 2025;23:e3003298. 40690527 10.1371/journal.pbio.3003298 PMC12306757

[pro70682-bib-0107] Rogne M , Landsverk HB , Van Eynde A , Beullens M , Bollen M , Collas P , et al. The KH‐Tudor domain of A‐kinase anchoring protein 149 mediates RNA‐dependent self‐association. Biochemistry. 2006;45:14980–14989. 17154535 10.1021/bi061418y

[pro70682-bib-0108] Saint‐Georges Y , Garcia M , Delaveau T , Jourdren L , Crom SL , Lemoine S , et al. Yeast mitochondrial biogenesis: a role for the PUF RNA‐binding protein Puf3p in mRNA localization. PLoS One. 2008;3:e2293. 18523582 10.1371/journal.pone.0002293 PMC2387061

[pro70682-bib-0109] Santos J , Günnigmann M , Gora RJ , Iljina M , Predin M , Kotan IE , et al. NAC promotes co‐translational protein folding at the ribosomal tunnel exit. Mol Cell. 2026;86:1311–1326.e11. 41875886 10.1016/j.molcel.2026.02.022 PMC13020645

[pro70682-bib-0110] Sass E , Karniely S , Pines O . Folding of fumarase during mitochondrial import determines its dual targeting in yeast. J Biol Chem. 2003;278:45109–45116. 12960177 10.1074/jbc.M302344200

[pro70682-bib-0111] Schibich D , Gloge F , Pöhner I , Björkholm P , Wade RC , von Heijne G , et al. Global profiling of SRP interaction with nascent polypeptides. Nature. 2016;536:219–223. 27487212 10.1038/nature19070

[pro70682-bib-0112] Schuller AP , Green R . Roadblocks and resolutions in eukaryotic translation. Nat Rev Mol Cell Biol. 2018;19:526–541. 29760421 10.1038/s41580-018-0011-4 PMC6054806

[pro70682-bib-0113] Sen A , Cox RT . Clueless is a conserved ribonucleoprotein that binds the ribosome at the mitochondrial outer membrane. Biol Open. 2016;5:195–203. 26834020 10.1242/bio.015313 PMC4823986

[pro70682-bib-0114] Sharma S , Fazal FM . Localization of RNAs to the mitochondria—mechanisms and functions. RNA. 2024;30:597–608. 38448244 10.1261/rna.079999.124 PMC11098466

[pro70682-bib-0115] Shiber A , Döring K , Friedrich U , Klann K , Merker D , Zedan M , et al. Cotranslational assembly of protein complexes in eukaryotes revealed by ribosome profiling. Nature. 2018;561:268–272. 30158700 10.1038/s41586-018-0462-y PMC6372068

[pro70682-bib-0116] Shiota T , Imai K , Qiu J , Hewitt VL , Tan K , Shen H‐H , et al. Molecular architecture of the active mitochondrial protein gate. Science. 2015;349:1544–1548. 26404837 10.1126/science.aac6428

[pro70682-bib-0117] Stein KC , Kriel A , Frydman J . Nascent polypeptide domain topology and elongation rate direct the cotranslational hierarchy of Hsp70 and TRiC/CCT. Mol Cell. 2019;75:1117–1130.e5. 31400849 10.1016/j.molcel.2019.06.036 PMC6953483

[pro70682-bib-0118] Sylvestre J , Vialette S , Corral Debrinski M , Jacq C . Long mRNAs coding for yeast mitochondrial proteins of prokaryotic origin preferentially localize to the vicinity of mitochondria. Genome Biol. 2003;4:R44. 12844360 10.1186/gb-2003-4-7-r44 PMC193631

[pro70682-bib-0119] Tanenbaum ME , Gilbert LA , Qi LS , Weissman JS , Vale RD . A protein tagging system for signal amplification in gene expression and fluorescence imaging. Cell. 2014;159:635–646. 25307933 10.1016/j.cell.2014.09.039 PMC4252608

[pro70682-bib-0120] Tsuboi T , Viana MP , Xu F , Yu J , Chanchani R , Arceo XG , et al. Mitochondrial volume fraction and translation duration impact mitochondrial mRNA localization and protein synthesis. Elife. 2020;9:e57814. 32762840 10.7554/eLife.57814 PMC7413667

[pro70682-bib-0121] Verma R , Reichermeier KM , Burroughs AM , Oania RS , Reitsma JM , Aravind L , et al. Vms1 and ANKZF1 peptidyl‐tRNA hydrolases release nascent chains from stalled ribosomes. Nature. 2018;557:446–451. 29632312 10.1038/s41586-018-0022-5 PMC6226276

[pro70682-bib-0122] Vögtle F‐N , Wortelkamp S , Zahedi RP , Becker D , Leidhold C , Gevaert K , et al. Global analysis of the mitochondrial N‐proteome identifies a processing peptidase critical for protein stability. Cell. 2009;139:428–439. 19837041 10.1016/j.cell.2009.07.045

[pro70682-bib-0123] von Heijne G . Mitochondrial targeting sequences may form amphiphilic helices. EMBO J. 1986;5:1335–1342. 3015599 10.1002/j.1460-2075.1986.tb04364.x PMC1166945

[pro70682-bib-0124] Voorhees RM , Fernández IS , Scheres SHW , Hegde RS . Structure of the mammalian ribosome‐Sec61 complex to 3.4 Å resolution. Cell. 2014;157:1632–1643. 24930395 10.1016/j.cell.2014.05.024 PMC4081569

[pro70682-bib-0125] Waltz F , Righetto RD , Lamm L , Salinas‐Giegé T , Kelley R , Zhang X , et al. In‐cell architecture of the mitochondrial respiratory chain. Science. 2025;387:1296–1301. 40112058 10.1126/science.ads8738

[pro70682-bib-0126] Wang S , Sakai H , Wiedmann M . NAC covers ribosome‐associated nascent chains thereby forming a protective environment for regions of nascent chains just emerging from the peptidyl transferase center. J Cell Biol. 1995;130:519–528. 7622554 10.1083/jcb.130.3.519 PMC2120527

[pro70682-bib-0127] Wiedmann B , Prehn S . The nascent polypeptide‐associated complex (NAC) of yeast functions in the targeting process of ribosomes to the ER membrane. FEBS Lett. 1999;458:51–54. 10518932 10.1016/s0014-5793(99)01118-7

[pro70682-bib-0128] Wiedmann B , Sakai H , Davis TA , Wiedmann M . A protein complex required for signal‐sequence‐specific sorting and translocation. Nature. 1994;370:434–440. 8047162 10.1038/370434a0

[pro70682-bib-0129] Williams CC , Jan CH , Weissman JS . Targeting and plasticity of mitochondrial proteins revealed by proximity‐specific ribosome profiling. Science. 2014;346:748–751. 25378625 10.1126/science.1257522 PMC4263316

[pro70682-bib-0130] Willmund F , del Alamo M , Pechmann S , Chen T , Albanèse V , Dammer EB , et al. The cotranslational function of ribosome‐associated Hsp70 in eukaryotic protein homeostasis. Cell. 2013;152:196–209. 23332755 10.1016/j.cell.2012.12.001 PMC3553497

[pro70682-bib-0131] Wrobel L , Topf U , Bragoszewski P , Wiese S , Sztolsztener ME , Oeljeklaus S , et al. Mistargeted mitochondrial proteins activate a proteostatic response in the cytosol. Nature. 2015;524:485–488. 26245374 10.1038/nature14951

[pro70682-bib-0132] Wu CC‐C , Peterson A , Zinshteyn B , Regot S , Green R . Ribosome collisions trigger general stress responses to regulate cell fate. Cell. 2020;182:404–416.e14. 32610081 10.1016/j.cell.2020.06.006 PMC7384957

[pro70682-bib-0133] Xia C , Fan J , Emanuel G , Hao J , Zhuang X . Spatial transcriptome profiling by MERFISH reveals subcellular RNA compartmentalization and cell cycle‐dependent gene expression. Proc Natl Acad Sci. 2019;116:19490–19499. 31501331 10.1073/pnas.1912459116 PMC6765259

[pro70682-bib-0134] Yamano K , Yatsukawa Y , Esaki M , Hobbs AEA , Jensen RE , Endo T . Tom20 and Tom22 share the common signal recognition pathway in mitochondrial protein import. J Biol Chem. 2008;283:3799–3807. 18063580 10.1074/jbc.M708339200

[pro70682-bib-0135] Yan X , Hoek TA , Vale RD , Tanenbaum ME . Dynamics of translation of single mRNA molecules in vivo. Cell. 2016;165:976–989. 27153498 10.1016/j.cell.2016.04.034 PMC4889334

[pro70682-bib-0136] Yogev O , Karniely S , Pines O . Translation‐coupled translocation of yeast fumarase into mitochondria in vivo. J Biol Chem. 2007;282:29222–29229. 17666392 10.1074/jbc.M704201200

[pro70682-bib-0137] Young JC , Hoogenraad NJ , Hartl FU . Molecular chaperones Hsp90 and Hsp70 deliver preproteins to the mitochondrial import receptor Tom70. Cell. 2003;112:41–50. 12526792 10.1016/s0092-8674(02)01250-3

[pro70682-bib-0138] Zaninello M , Schlegel T , Nolte H , Pirzada M , Savino E , Barth E , et al. CLUH maintains functional mitochondria and translation in motoneuronal axons and prevents peripheral neuropathy. Sci Adv. 2024;10:eadn2050. 38809982 10.1126/sciadv.adn2050 PMC11135423

[pro70682-bib-0139] Zhang Y , Chen Y , Gucek M , Xu H . The mitochondrial outer membrane protein MDI promotes local protein synthesis and mtDNA replication. EMBO J. 2016;35:1045–1057. 27053724 10.15252/embj.201592994 PMC4868955

[pro70682-bib-0140] Zhang Y , Valentín Gesé G , Conz C , Lapouge K , Kopp J , Wölfle T , et al. The ribosome‐associated complex RAC serves in a relay that directs nascent chains to Ssb. Nat Commun. 2020;11:1504. 32198371 10.1038/s41467-020-15313-w PMC7083937

[pro70682-bib-0141] Zhao T , Chen Y‐M , Li Y , Wang J , Chen S , Gao N , et al. Disome‐seq reveals widespread ribosome collisions that promote cotranslational protein folding. Genome Biol. 2021;22:16. 33402206 10.1186/s13059-020-02256-0 PMC7784341

[pro70682-bib-0142] Zhou X , Yang Y , Wang G , Wang S , Sun D , Ou X , et al. Molecular pathway of mitochondrial preprotein import through the TOM–TIM23 supercomplex. Nat Struct Mol Biol. 2023;30:1996–2008. 37696957 10.1038/s41594-023-01103-7

[pro70682-bib-0143] Zhu X , Cruz VE , Zhang H , Erzberger JP , Mendell JT . Specific tRNAs promote mRNA decay by recruiting the CCR4‐NOT complex to translating ribosomes. Science. 2024;386:eadq8587. 39571015 10.1126/science.adq8587 PMC11583848

[pro70682-bib-0144] Zhu Z , Mallik S , Stevens TA , Huang R , Levy ED , Shan S . Principles of cotranslational mitochondrial protein import. Cell. 2025;188:5605–5617.e14. 40795856 10.1016/j.cell.2025.07.021 PMC12396113

[pro70682-bib-0145] Zilio E , Schlegel T , Zaninello M , Rugarli EI . The role of mitochondrial mRNA translation in cellular communication. J Cell Sci. 2025;138:jcs263753. 40326563 10.1242/jcs.263753

[pro70682-bib-0146] Zurita Rendón O , Fredrickson EK , Howard CJ , Van Vranken J , Fogarty S , Tolley ND , et al. Vms1p is a release factor for the ribosome‐associated quality control complex. Nat Commun. 2018;9:2197. 29875445 10.1038/s41467-018-04564-3 PMC5989216

